# A road map from single-cell transcriptome to patient classification for the immune response to trauma

**DOI:** 10.1172/jci.insight.145108

**Published:** 2021-01-25

**Authors:** Tianmeng Chen, Matthew J. Delano, Kong Chen, Jason L. Sperry, Rami A. Namas, Ashley J. Lamparello, Meihong Deng, Julia Conroy, Lyle L. Moldawer, Philip A. Efron, Patricia Loughran, Christopher Seymour, Derek C. Angus, Yoram Vodovotz, Wei Chen, Timothy R. Billiar

**Affiliations:** 1 Department of Surgery, University of Pittsburgh, Pittsburgh, Pennsylvania, USA.; 2 Cellular and Molecular Pathology program, Department of Pathology, University of Pittsburgh School of Medicine, Pittsburgh, Pennsylvania, USA.; 3 Department of Surgery, University of Michigan, Ann Arbor, Michigan, USA.; 4 Department of Medicine, University of Pittsburgh, Pittsburgh, Pennsylvania, USA.; 5 Department of Surgery, University of Florida College of Medicine, Gainesville, Florida, USA.; 6 The Clinical Research, Investigation and Systems Medicine of Acute Illness (CRISMA) Center, Department of Critical Care Medicine, University of Pittsburgh, Pittsburgh, Pennsylvania, USA.; 7 Department of Pediatrics, University of Pittsburgh, Pittsburgh, Pennsylvania, USA.

**Keywords:** Immunology, Inflammation, Bioinformatics, Innate immunity, Surgery

## Abstract

Immune dysfunction is an important factor driving mortality and adverse outcomes after trauma but remains poorly understood, especially at the cellular level. To deconvolute the trauma-induced immune response, we applied single-cell RNA sequencing to circulating and bone marrow mononuclear cells in injured mice and circulating mononuclear cells in trauma patients. In mice, the greatest changes in gene expression were seen in monocytes across both compartments. After systemic injury, the gene expression pattern of monocytes markedly deviated from steady state with corresponding changes in critical transcription factors, which can be traced back to myeloid progenitors. These changes were largely recapitulated in the human single-cell analysis. We generalized the major changes in human CD14^+^ monocytes into 6 signatures, which further defined 2 trauma patient subtypes (SG1 vs. SG2) identified in the whole-blood leukocyte transcriptome in the initial 12 hours after injury. Compared with SG2, SG1 patients exhibited delayed recovery, more severe organ dysfunction, and a higher incidence of infection and noninfectious complications. The 2 patient subtypes were also recapitulated in burn and sepsis patients, revealing a shared pattern of immune response across critical illness. Our data will be broadly useful to further explore the immune response to inflammatory diseases and critical illness.

## Graphical abstract

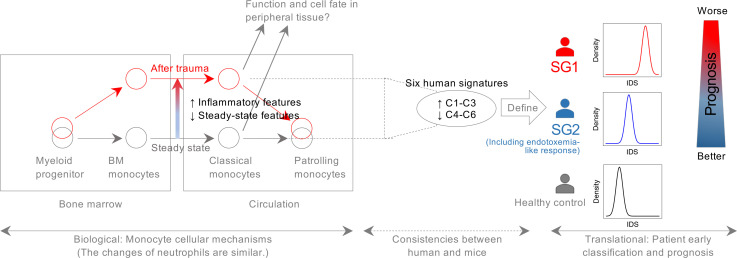

## Introduction

Trauma causes an abrupt transition from a healthy state (i.e., homeostasis) to a state best described as a system-wide physiological crisis. Severe injury is so common among humans that it is the leading cause of death and morbidity in individuals under 54 years old. Advances in clinical management have reduced early deaths substantially; however, persistent organ dysfunction and delayed infections, both associated with immune dysfunction, remain poorly understood and difficult to prevent (1, 2).

Insights into the mechanisms leading to immune dysfunction after trauma have lagged behind other diseases associated with a disordered immune response. Transcriptomic analysis of unseparated circulating leukocytes from severely injured humans revealed a “genomic storm” with more than 80% of the leukocyte transcriptome altered during the first 28 days after systemic injury (3). That study introduced a novel paradigm to describe the immune-inflammatory response to trauma: an early induction of excessive proinflammatory pathways and simultaneous suppression of adaptive immune responses. Patients suffering complicated courses manifested leukocyte transcriptional patterns consistent with prolonged immune dysregulation (3). Cabrera et al. demonstrated that differential transcriptomic changes could be identified within whole-blood leukocytes within 2 hours in severely injured patients who subsequently developed multiple organ dysfunction syndrome (4). Studies at the single-cell level have been limited to the identification of the appearance of Th17 cells by mass cytometry (CyTOF) in the circulation of severely injured patients (5). Thus, little is known about the cell-specific pathways behind the pathogenic inflammation and immunosuppression that follow trauma.

To provide the landscape of transcriptomic changes at the single-cell level after systemic injury, we carried out single-cell RNA sequencing (scRNA-Seq) on bone marrow and/or circulating mononuclear cells from injured mice and humans. Studies in both a well-controlled mouse model of trauma and a detailed time course study in 10 severely injured humans identified the greatest changes in Ly6C^+^/CD14^+^ monocytes. This led us to characterize the major regulatory features in myeloid cells after systemic injury. To correlate these features with outcomes, we analyzed databases representing global gene expression changes in circulating leukocytes in large patient studies of trauma, burns, and sepsis. In addition to providing a comprehensive landscape of the dynamic changes in transcriptomic patterns in myeloid mononuclear cells after severe injury, we identify patient subtypes with potential prognostic value along with the critical regulatory networks (transcription factors) at the cellular level.

## Results

### Dramatic transcriptomic changes in mouse circulating monocytes after systemic injury.

**Figure 1:**
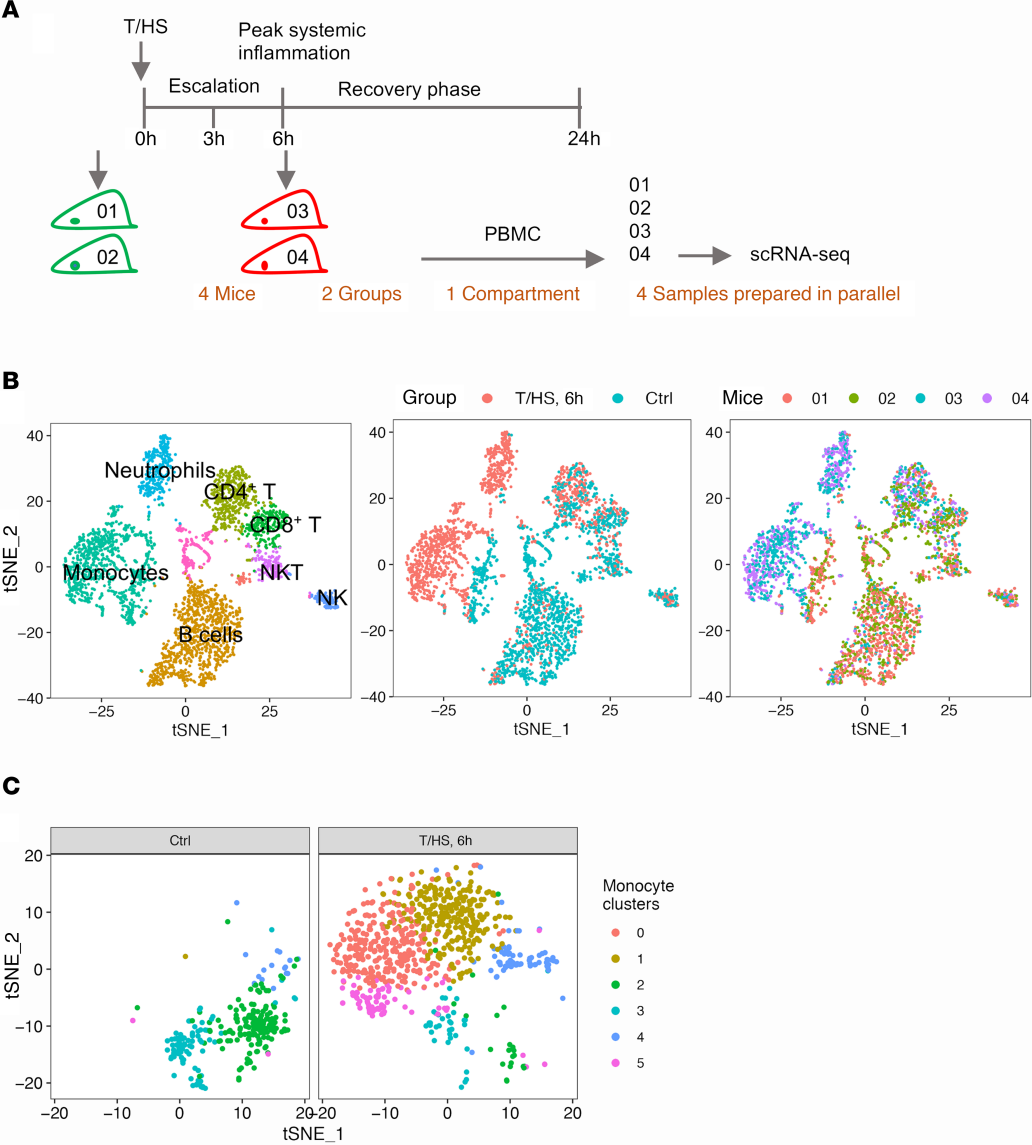
Overview of transcriptomic changes in mouse PBMCs at 6 hours after T/HS. (**A**) Experimental design of the data shown in Figure 1 and Figure 2 and Supplemental Figure 1 and Supplemental Figure 2, with 2 mice/group. (**B**) The t-SNE plot shows PBMCs from 4 mice color-coded by major cell types, by animal groups, or by individual mice. (**C**) The identified 6 clusters in circulating monocytes.

To deconvolute the immune response to trauma, scRNA-Seq was performed on peripheral blood mononuclear cells (PBMCs) isolated from mice subjected to tissue trauma with hemorrhagic shock (T/HS) (6, 7) and their uninjured littermates (2 mice/group) (Figure 1A). As shown in the t-distributed stochastic neighbor embedding (t-SNE) (8) plots (Figure 1B and Supplemental Figure 1A; supplemental material available online with this article; https://doi.org/10.1172/jci.insight.145108DS1), biological duplicates overlapped very well. Thus, the shift between experimental groups can be expected to reflect the trauma-induced differences. At 6 hours after injury, the peak of systemic inflammation (9), the greatest changes were observed in monocytes represented by an obvious transcriptomic shift in the t-SNE plot (Figure 1B) and the largest number of differentially expressed genes (DEGs) (Supplemental Figure 1, B–E).

**Figure 2:**
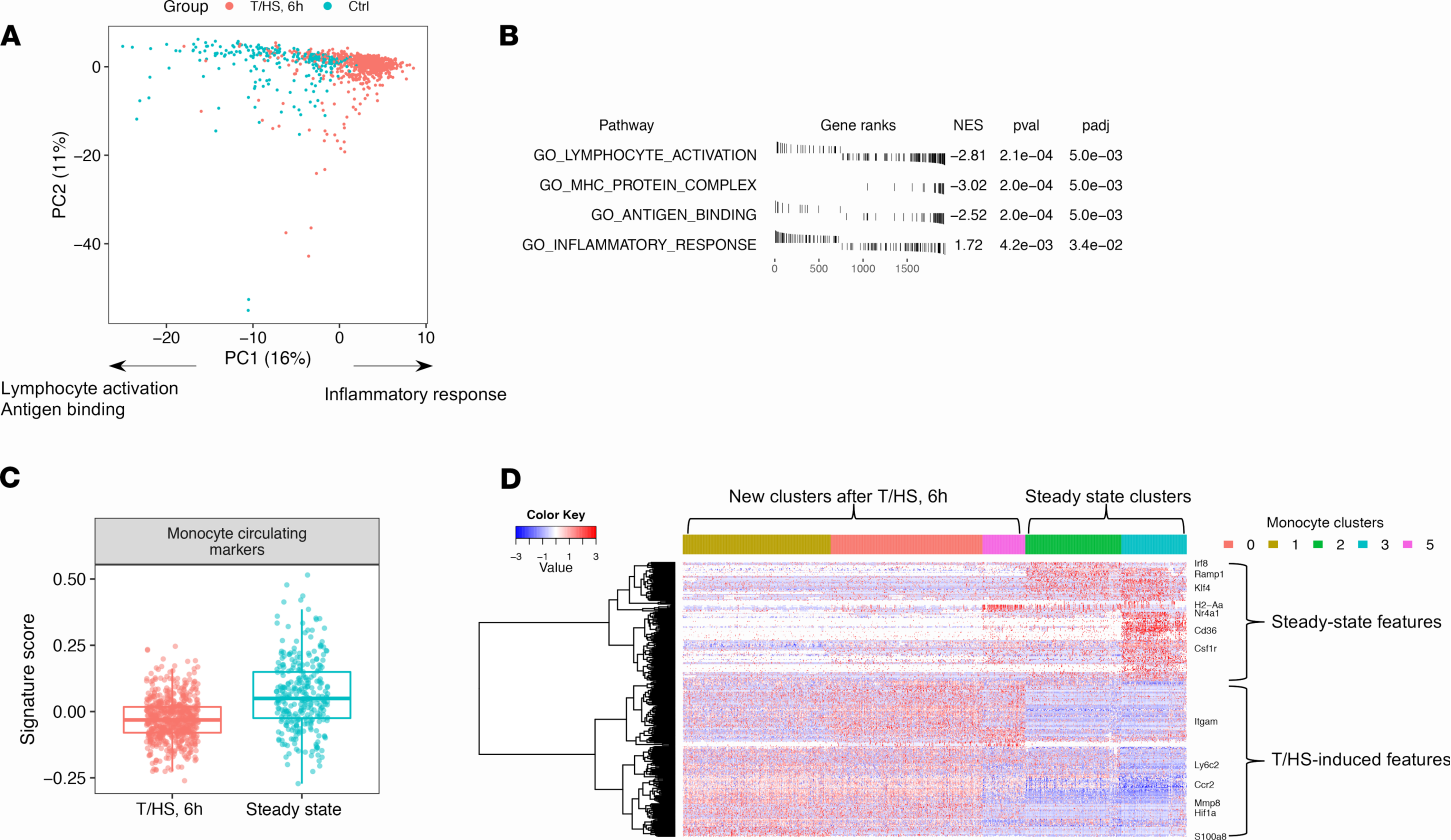
Dramatic transcriptomic changes in mouse circulating monocytes after systemic injury. (**A**) PCA plot of circulating monocytes color-coded by groups. Biological interpretations are annotated based on the results shown in **B**. (**B**) Selected enriched gene ontology (GO) terms of PC1-associated genes by GSEA. (**C**) The monocytes after T/HS express fewer monocyte circulating markers than monocytes from control mice. The boxes span from the Q1 to the Q3, with the center line showing the median. Lower whiskers represent Q1 – 1.5*IQR, and upper whiskers represent Q3 + 1.5*IQR (Q1, the first quantile; Q3, the third quantile; IQR = Q3 – Q1). (**D**) RNA profile of circulating monocytes built upon pairwise DEGs between each 2-cluster combination. Cells (columns) are ordered by clusters. Genes (rows) are clustered into 2 large clusters generally representing either steady-state or T/HS-induced features. Single-cell transcriptomic data were collected from *n* = 2 mice/group as shown in Figure 1A.

To characterize monocytes at a higher resolution, we extracted and reanalyzed monocytes separately. The designation of mouse circulating monocytes is based on the surface marker Ly6C (coded by *Ly6c2*) (10), and circulating Ly6C^+^ monocytes give rise to Ly6C^–^ monocytes (11). We recapitulated 2 steady-state clusters corresponding to classical (cluster 2: *Ly6c2*^+^) and patrolling monocytes (cluster 3: *Ly6c2*^–^) (10) in the uninjured mice. We also identified 3 new monocyte clusters that were distinct from steady-state monocytes, which showed a gradient in *Ly6c2* expression after T/HS (clusters 1, 0, and 5: *Ly6c2*^hi^, *Ly6c2*^int^, *Ly6c2*^lo^). Cluster 4 comprised monocyte-platelet aggregates highly expressing platelet markers (*Pf4* and *Pbpp*) (Figure 1C and Supplemental Figure 2A). Principal component analysis (PCA) revealed a right shift of monocytes after T/HS on principal component 1 (PC1). Based on gene set enrichment analysis (GSEA) (12), the right side of PC1 associated with inflammation and the left side with lymphocyte activation (Figure 2, A and B), suggesting that T/HS-induced monocytes are more inflammatory but deficient in the capacity for lymphocyte activation compared with steady-state monocytes.

The monocyte clusters that appeared after T/HS could be derived from cells already present in the circulation that underwent transcriptional changes or from bone marrow (BM). To address these possibilities, we generated a customized gene signature representing the upregulated genes in circulating monocytes compared with BM monocytes under steady state (11) (Supplemental Figure 2, B and C). Monocyte-platelet aggregates (cluster 4) were excluded from the analyses of developmental status because of the confounding effects of multiplets in single-cell analysis. The newly identified monocytes after T/HS displayed lower signature scores than the steady-state monocytes (Figure 2C), indicating that the monocytes after T/HS were more immature. Furthermore, the DEGs were largely preserved in the newly identified clusters after T/HS (1→0→5, “→” followed a decreasing gradient in *Ly6c2* expression) and in the steady-state clusters (2→3) but were minimally shared between the 2 experimental conditions (Figure 2D). Both of these observations suggested that the new monocyte clusters observed after trauma were derived from BM.

### Continuous changes in the myeloid cell transcriptome from the BM to the circulation after T/HS.

**Figure 3:**
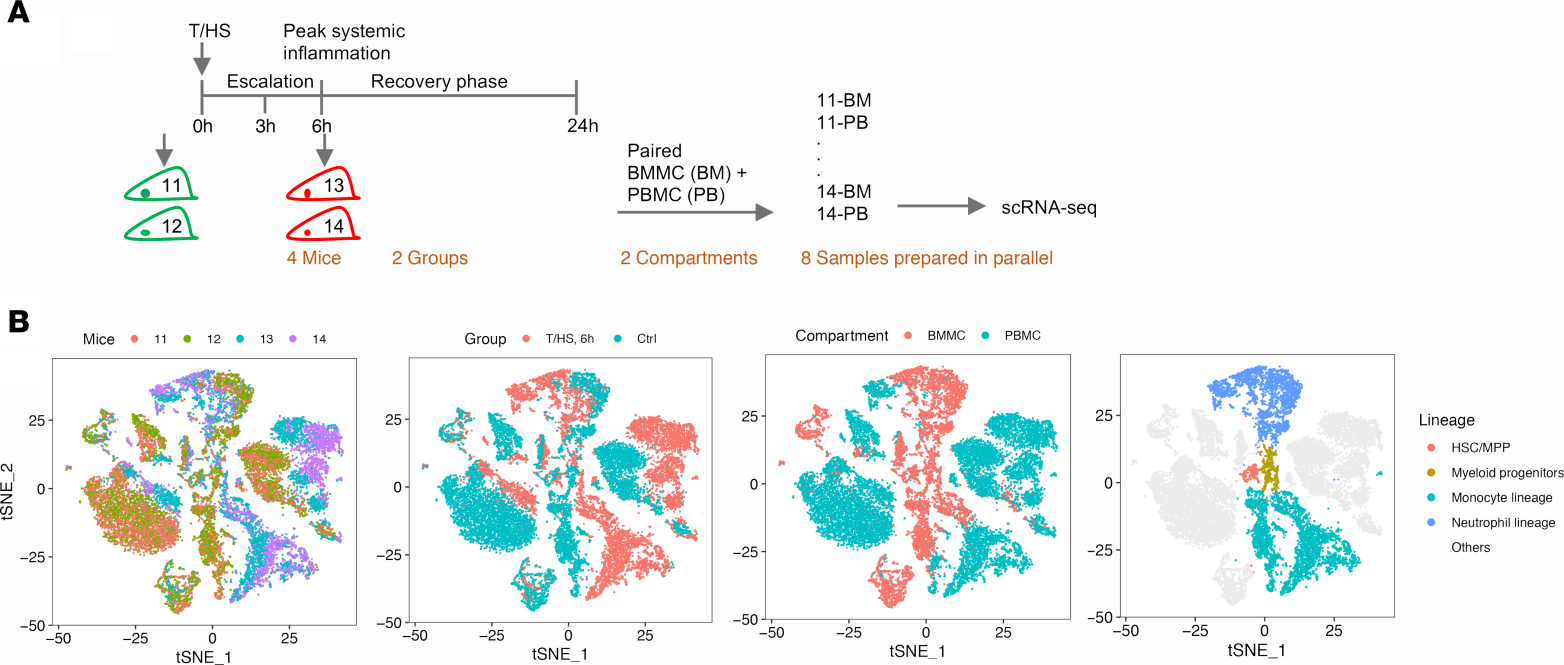
Overview of paired mouse BMMCs and PBMCs at 6 hours after T/HS. (**A**) Experimental design for the data shown in Figures 3–7 and Supplemental Figures 3–6, with 2 mice/group. (**B**) The t-SNE plot of BMMC + PBMC from the 4 mice color-coded by individual mice, by groups, by cell compartments, or by cell lineages. HSC, hematopoietic stem cell; MPP, multipotent progenitor.

**Figure 4:**
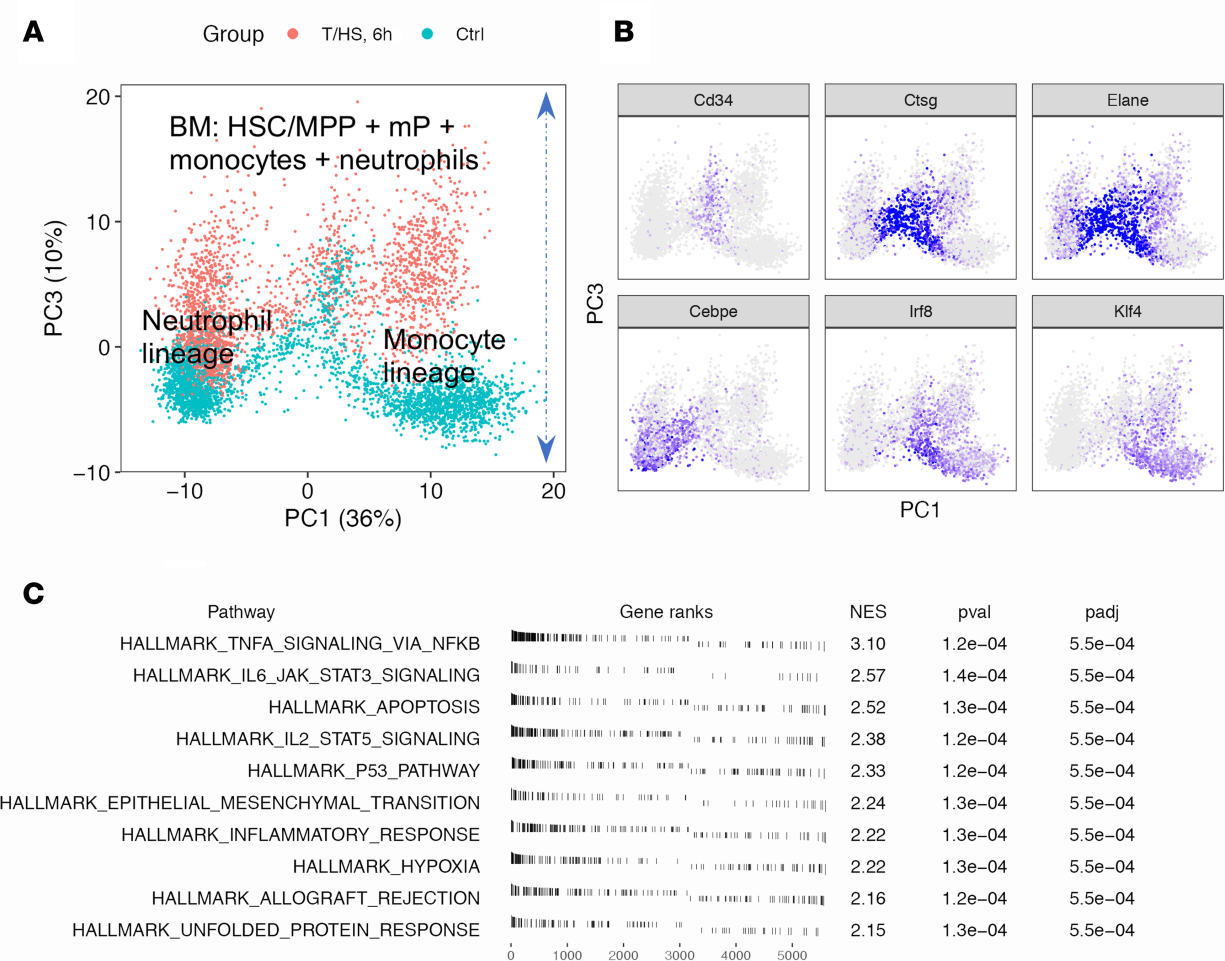
Overview of transcriptomic changes in BM myeloid cells at 6 hours after T/HS. (**A**) PCA plot of BM myeloid cells including hematopoietic stem cells/multipotent progenitors (HSC/MPP) and myeloid progenitors (mPs) as shown in the fourth panel of Figure 3B color-coded by groups. (**B**) Expression of lineage markers. (**C**) Top 10 hallmark pathways enriched on the positive side of PC3. Single-cell transcriptomic data were collected from *n* = 2 mice/group as shown in Figure 3A.

We next carried out scRNA-Seq on paired PBMC and BM mononuclear cells (BMMCs) from additional control and T/HS mice at 6 hours (2 mice/group) (Figure 3A). t-SNE across the circulating and BM compartments displayed large differences in myeloid cells after T/HS, especially in the monocyte lineage. The changes initiated in the BM were continuous to the circulating compartment (Figure 3B and Supplemental Figure 3A). PCA indicated that BM neutrophil and monocyte lineages underwent similar changes after systemic injury demonstrated by the positive side of PC3 and represented by inflammation, response to stress, and apoptosis (Figure 4, A–C). The circulating monocytes from this batch reproduced the major changes from the first experiment (Supplemental Figure 3, B and C).

The regulatory pathways associated with the myeloid trajectories were further explored by computing gene regulatory networks (regulons) using SCENIC (13). A regulon represents the coexpressed set of genes detected within scRNA-Seq data, including a core transcription factor (TF) and the TF regulated genes containing the TF binding motif depicted as “TF (number of genes).” We followed published methods (14, 15) and projected the regulons on PCA 2D space. Well-established TFs largely overlay with the known corresponding lineages (Supplemental Figure 3, D–F), supporting the reliability of the computed regulons. SCENIC provides 2 kinds of regulons: (a) main regulons (unextended) only using the high-confidence annotations and (b) extended regulons also including lower confidence annotations. Both types of regulons yielded similar results (Supplemental Figure 3F). In the following analysis, we only used the main regulons to establish the regulatory landscape.

### Characterization of the transcriptomic changes in myeloid progenitors after T/HS.

**Figure 5:**
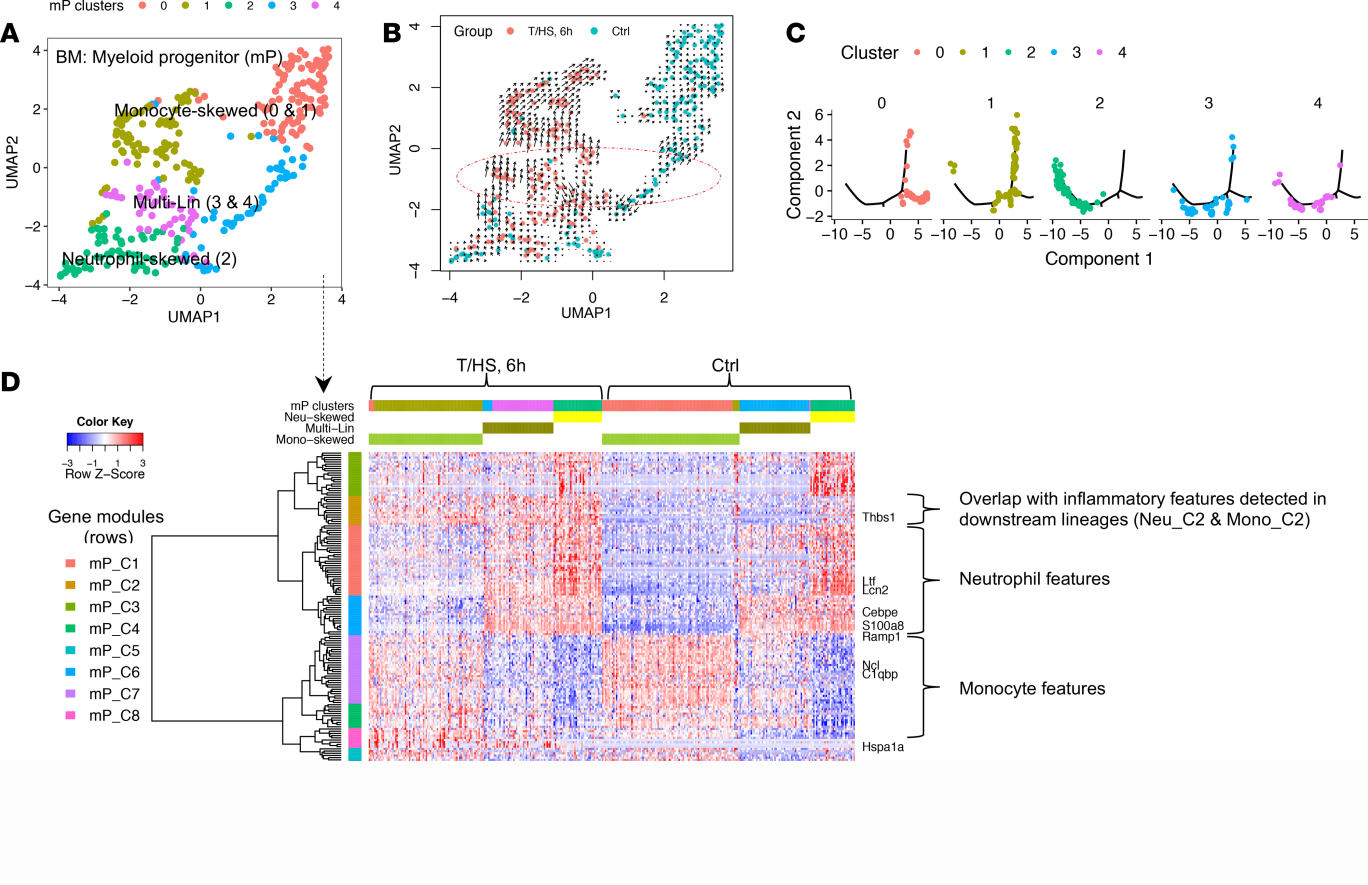
Characterization of transcriptomic changes in the BM mPs at 6 hours after T/HS. (**A**) Uniform Manifold Approximation and Projection (UMAP) plot color-coded by 5 mP clusters. (**B**) UMAP plot color-coded by groups (RNA velocity shown as arrows in the circled region). (**C**) The developmental trajectories constructed by Monocle 2. Cells are color-coded by mP clusters. (**D**) RNA profile built upon pairwise DEGs between mP clusters (fold change ≥ 2 and Bonferroni-adjusted *P* < 0.05). Cells (columns) are ordered first by groups, then by clusters. Genes (rows) are clustered into 8 gene modules (mP_C1~C8). Single-cell transcriptomic data were collected from *n* = 2 mice/group as shown in Figure 3A.

We next characterized the transcriptomic changes in myelopoiesis at a proximal branching point by analyzing BM mPs from control and T/HS mice (Figure 3B, the fourth panel). These cells coexpressed mP mRNA markers (*Ctsg*, *Mpo*, and *Elane*) (Supplemental Figure 3, A and G) and largely corresponded to common myeloid progenitors and granulocyte-monocyte progenitors as previously characterized (16). We identified 5 distinct progenitor clusters (Figure 5A) that included multi-Lin (clusters 3 and 4), monocyte-skewed (clusters 0 and 1), and neutrophil-skewed (cluster 2) mPs as shown in UMAP (17). RNA velocity (18) is an algorithm estimating the future state of single cells. The opposite directions in the RNA velocity (shown by the arrows in the circled region) observed for clusters 3 and 4 further suggest 2 potential cell fates and support their identity as multi-Lin progenitors (Figure 5B). Monocle (19) is an algorithm to reconstruct the trajectory of differentiation using scRNA-Seq data. The trajectories of mP established by Monocle (Figure 5C) were largely consistent with the identified clusters.

The transcriptomic profiles between the 5 mP clusters were established by pairwise DEG comparisons. Ward’s hierarchical clustering (20) yielded 8 gene modules (mP_C1–C8) (Figure 5D). All mouse gene modules in this study are annotated as “CellType_Cluster (C)#.” The steady-state multi-Lin mP expressed neutrophil and monocyte lineage modules at low levels, while clusters representing skewed mPs highly expressed single lineage-specific modules. These patterns are consistent with the binary cell fate choice in mPs described under baseline conditions by Olsson et al. (16). Trauma induced 2 major changes that include (a) a monocyte-to-neutrophil shift in multi-Lin mPs and (b) an upregulation of gene module mP_C2 across all T/HS mP clusters. The features of mP_C2 were preserved in the analysis of the downstream monocytes and neutrophils discussed below (Supplemental Figure 3H).

### Characterization of the transcriptomic changes in the BM monocyte lineage after T/HS.

**Figure 6:**
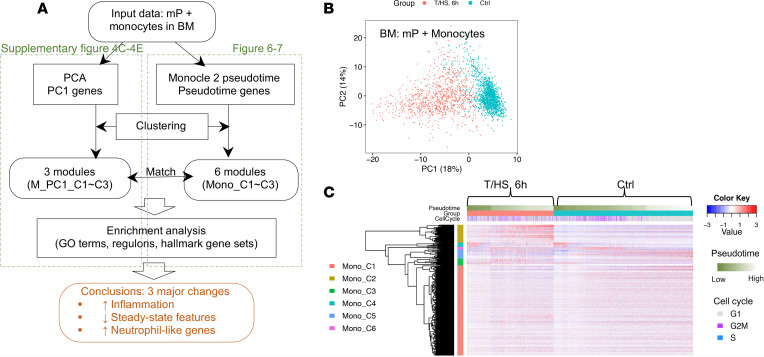
Overview of transcriptomic changes in the BM monocyte lineages at 6 hours after T/HS. (**A**) Schema describing the workflow for Figure 6 and Figure 7 and Supplemental Figure 4 and Supplemental Figure 5. (**B**) PCA plot of BM monocyte lineage (including all mPs, as shown in the fourth panel of Figure 3B) color-coded by groups. (**C**) RNA profile of the BM monocyte lineage built upon pseudotime-associated genes identified by Monocle 2. Cells (columns) are ordered first by groups, then by pseudotime. Genes (rows) are clustered into 6 gene modules (Mono_C1~C6) as shown in (Table 1). Single-cell transcriptomic data were collected from *n* = 2 mice/group as shown in Figure 3A.

To characterize the full developmental trajectories during monopoiesis, we analyzed BM monocytes along with BM mPs together (scheme of the analysis depicted in Figure 6A). PCA indicated that T/HS induced dramatic changes in the monocyte lineage represented by PC1 (Figure 6B and Supplemental Figure 4A). To characterize the changes along monocyte differentiation, we first computed pseudotime using Monocle 2 (19). The pseudotime analysis was validated using genes extracted from an independent data set that correlated with normal BM monocyte differentiation (11). In control mice, the changes of these genes were consistent with our computed pseudotime, confirming that our pseudotime is biologically relevant (Supplemental Figure 4B).

**Figure 7:**
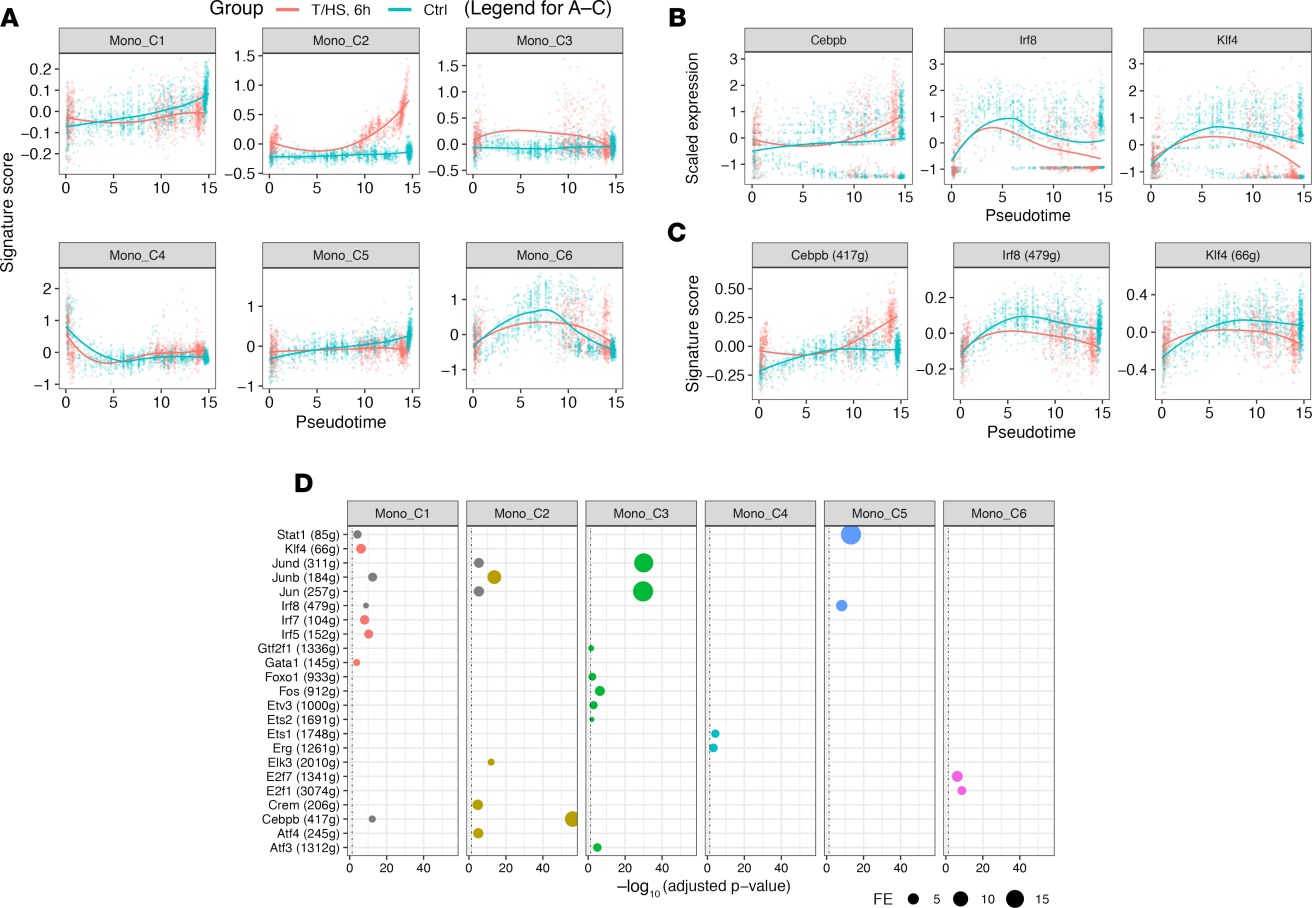
Characterization of transcriptomic changes in the BM monocyte lineages at 6 hours after T/HS. (**A**–**C**) Expression of each gene module (**A**), critical TF (**B**), and corresponding regulon (**C**) along pseudotime. Smoothing lines were fitted by Loess regression. (**D**) Enrichment analysis between gene modules and regulons. Hypergeometric *P* value was computed. Only the relationships with Benjamini-Hochberg–adjusted *P* < 0.05 (labeled as black, vertical, dashed line) with fold enrichment (FE) ≥ 2 and the number of overlapping genes ≥ 15 are shown. Relationships are color-coded by top enriched gene modules (with highest FE). Single-cell transcriptomic data were collected from *n* = 2 mice/group as shown in Figure 3A. Klf4, Kruppel-like factor 4; Irf8, IFN regulatory factor 8.

We next established the RNA profile of pseudotime-associated genes and identified 6 gene modules (Mono_C1–C6) by Ward’s clustering (Figure 6C). To interpret biological functions and reveal critical regulatory networks, we performed an enrichment analysis using the molecular signatures database (MSigDB) (21) GO and hallmark gene sets and computed regulons using SCENIC (13) (Table 1 and Figure 7, A–D). Mono_C2, which was *Cebpb* (C/EBPβ protein coding gene) regulon associated, was upregulated after T/HS and increased continuously along differentiation (Figure 7A). This is consistent with the known roles for *Cebpb* in emergency myelopoiesis (22, 23). The CD11b coding gene, *Itgam*, was also included in Mono_C2 (Table 1), and this corresponded to changes observed in circulating cells (Figure 2D). *Klf4* and *Irf8* are TFs critical for steady-state monopoiesis (24). *Irf8* is also responsible for monocyte lineage commitment (16). Mono_C1 and Mono_C5 were associated with *Klf4* and *Irf8* regulons, respectively (Figure 7D). The monocyte lineage marker *Csf1r* (gene coding CD115) was included in Mono_C1 (Table 1). Unexpectedly, both Mono_C1 and Mono_C5 were downregulated after T/HS (Figure 7A). The changes in the gene expression of these critical TFs after T/HS were consistent with the corresponding changes in regulon expression (Figure 7, B and C), further supporting the results of the regulon enrichment analysis. We also identified a cell cycle module (Mono_C6), a progenitor module (Mono_C4), and a stress-responsive module (Mono_C3) (Table 1 and Figure 7).

To characterize the major differences in BM monocytes between control and T/HS, we extracted PC1-associated genes from the PCA shown in Figure 6B (Pearson’s correlation: adjusted *P* < 0.05 and |*r*| ≥ 0.3) and identified 3 gene modules (MonoPC1_C1–C3) (Figure 6A and Supplemental Figure 4C). MonoPC1_C1 corresponded to steady-state module Mono_C5. MonoPC1_C2 related to the inflammatory module found in Mono_C2 and mP_C2. In addition, we identified a neutrophil-like module MonoPC1_C3 (e.g., *S100a8*, *S100a9*, *Ltf*, *Lcn2*) highest expressed in neutrophils and upregulated in monocyte lineage after T/HS, which is consistent with the monocyte-to-neutrophil shift observed in multi-Lin mP and can be mapped to mP_C1 (Supplemental Figure 4, D and E). These 3 modules derived from BM monocytes were also reflected in the circulating monocytes (Supplemental Figure 4F).

We validated the unexpected changes in 2 critical markers (monocyte lineage marker CD115/*Csf1r* and lineage TF *Irf8*) at the protein level in BM monocytes. Because CD115 gene expression was suppressed after T/HS, we used 2 gating strategies when selecting the cells in the monocyte lineage that included (a) Lin^–^CD115^+^Ly6G^–^ and (b) Lin^–^Ly6C^++^Ly6G^–^ (Supplemental Figure 5A). In control mice, the cells gated by the 2 strategies appeared identical. Following T/HS, the proportion of Lin^–^CD115^+^Ly6G^–^–defined monocytes was significantly lower than that of Lin^–^Ly6C^++^Ly6G^–^–defined monocytes because of a marked decrease in CD115 protein expression. Lin^–^Ly6C^++^Ly6G^–^ gated cells expressed less IRF8 and became more CD11b^+^. To visualize the colocalization of protein changes in Lin^–^Ly6C^++^Ly6G^–^ gated cells, we chose Matlab/Cyt3 (25). Similar to scRNA-Seq, monocytes from the 2 experimental groups were largely separated in 2D reduction space (first panel in Supplemental Figure 5B). The higher CD11b expression and suppressed protein levels of IRF8 and CD115 were colocalized in most of the monocytes after T/HS (circled area in Supplemental Figure 5B). Combined, the findings from the transcriptomics, regulatory networks, and multidimensional protein analyses consistently characterized known and potentially novel features in T/HS-induced monocytes compared with steady-state monocytes. Therefore, circulating monocytes after T/HS are not a simply an immature version of steady state but instead a new myeloid trajectory evident at the mP level.

### Characterization of the transcriptomic changes in the BM neutrophil lineage after T/HS.

To characterize the full developmental trajectory during emergency neutropoiesis, BM mPs and BM neutrophils were analyzed together (Supplemental Figure 6, A and B). Using the same scheme outlined in Figure 6A, we identified 4 gene modules (Neu_C1–C4) with differential expression along pseudotime (Supplemental Figure 6C). Similar to emergency monopoiesis, Neu_C2 was enriched in *Cebpb* regulons and associated with inflammation. After T/HS, this module continued to increase during neutrophil differentiation (Supplemental Figure 6, D–F). The well-established granulocyte-lineage TF *Cebpe* (16) and surface marker *Ly6g* were associated with Neu_C1. Even though *Cebpe* mRNA and regulon expression trended downward after T/HS (Supplemental Figure 6, G and H), the steady-state module represented by Neu_C1 was not obviously suppressed (Supplemental Figure 6F). The surface expression of Ly6G in BM neutrophils was also comparable before and after T/HS (Supplemental Figure 5A). This is in contrast to monocytes, where the steady-state features were obviously suppressed after T/HS.

Monocle (19) identified 3 states in the BM neutrophil lineage. State 3 corresponded to the progenitor state, and the other 2 states were neutrophil-committed states. Steady-state BM neutrophils were mostly in state 1. After T/HS, the proportion of the cells in state 2 increased dramatically, with only a small group of cells remaining in state 1 (Supplemental Figure 6, C and I). State 2 was characterized by high expression of the inflammatory module Neu_C2 (Supplemental Figure 6J). Thus, we defined state 2 as the stimulated state and state 1 as the unstimulated state. T/HS induced a marked shift to the stimulated state during neutropoiesis.

Taken together, our analysis of monocytes and neutrophils after T/HS in mice indicates that these cells become more inflammatory, represented as the upregulation of the inflammatory modules Mono_C2 and Neu_C2. Compared with neutrophils, monocytes displayed an earlier BM branching point and more pronounced changes, characterized as partially losing steady-state features and gaining neutrophil-associated genes. We also assessed for the presence of transcriptomic signatures described for myeloid-derived suppressor cells (MDSCs) (26) and found these to be simultaneously upregulated in both monocyte and neutrophil lineages after trauma (Supplemental Figure 7). The gene modules and changes in TFs derived from the 6-hour time point in mouse BM were present at 3 hours and partially preserved to the 24-hour time point following injury (Supplemental Figure 8).

### Overview of the time-dependent transcriptomic changes in PBMCs from trauma patients.

**Figure 8:**
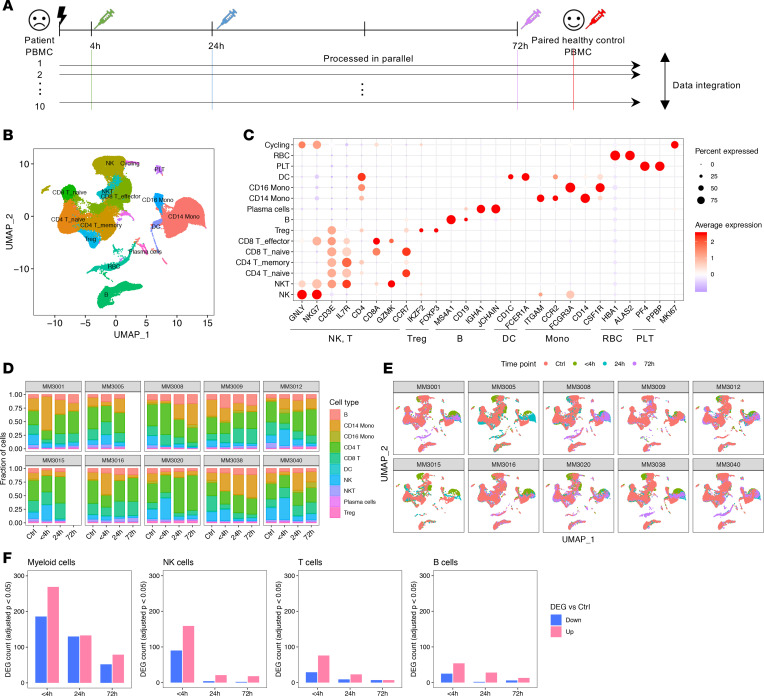
Overview of the transcriptomic changes in PBMCs from trauma patients over time. (**A**) Experimental design for human scRNA-Seq experiments. Blood samples for PBMC isolation were obtained within 4 hours of injury and at 24 hours and 72 hours after injury from 10 patients. Blood drawn from a healthy age- and sex-matched uninjured volunteer was used to establish the baseline for each patient. The 72-hour samples from 2 patients are not available, for a total of 38 samples (Ctrl: *n* = 10; <4 hours: *n* = 10; 24 hours: *n* = 10; 72 hours: *n* = 8). (**B**) UMAP plot of all human PBMCs are color-coded by major cell types. (**C**) Expression of major lineage markers in each cell type shown in **B**. (**D**) Changes of cell type composition in each patient along with matched control subject. (**E**) UMAP plot as shown in **B** wrapped by patients and color-coded by time points. (**F**) The number of significant DEGs (compared with healthy control, Bonferroni-adjusted *P* < 0.05) at different time points in major cell types.

To extend our landscape analysis into the human response to injury, we isolated PBMCs from 10 trauma patients (Supplemental Table 1) sampled at 3 time points (<4 hours, 24 hours, 72 hours) after systemic injury. Each patient was paired with an age- and sex-matched healthy control subject (Figure 8A). The 72-hour samples from 2 patients were not available, leaving 38 samples subjected to scRNA-Seq. A total of 151,470 immune cells passed initial quality control, with a mean of 1757 genes/cell.

The common PBMC cell types were easily distinguished in all 38 samples with clear differences in transcriptomic patterns (Figure 8, B and C). The frequencies of major cell types in healthy controls were consistent with a previous report (27). After trauma, changes in cell frequencies were variable between patients (Figure 8D). However, similar to mouse data, the myeloid compartment (mostly monocytes) exhibited a marked and time-dependent shift shown by UMAP (Figure 8E) and contained the largest number of DEGs among the major cell types (Figure 8F). NK cells displayed the second largest number of DEGs followed by lymphocytes. The greatest changes were observed at the <4-hour time point.

### Characterization of the transcriptomic changes in human circulating monocytes.

**Figure 9:**
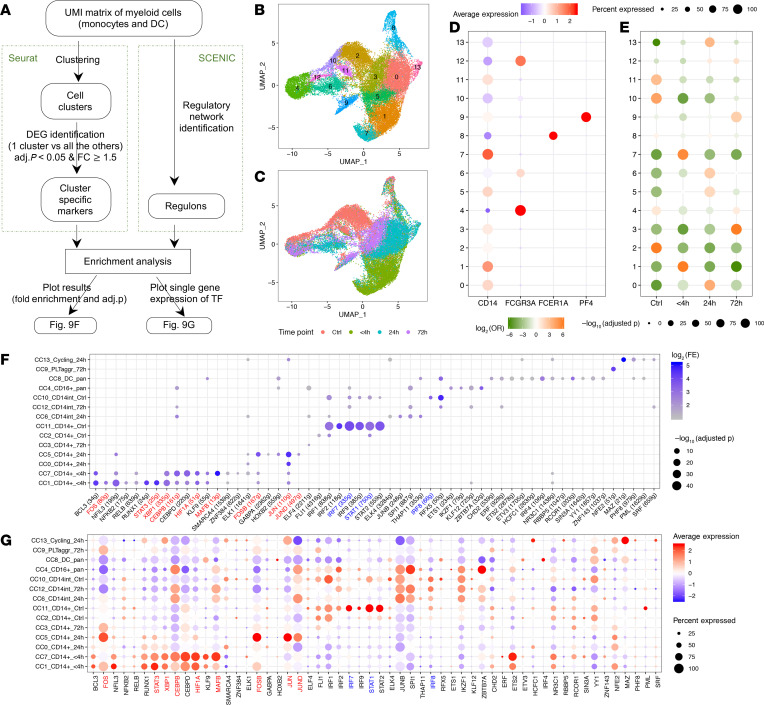
Characterization of the transcriptomic changes in human circulating monocytes after trauma. (**A**) Schema describing the workflow for **B**–**G**. Single-cell transcriptomic data were collected from 38 samples harvested at 4 different time points as shown in Figure 8A. UMI, unique molecular identifier. (**B** and **C**) UMAP plot of all human peripheral blood mononuclear myeloid cells color-coded by identified clusters (**B**) or by time points (**C**). (**D**) Expression of monocyte, DC, and platelet representative markers. (**E**) Overlap between myeloid clusters and time points was evaluated by χ^2^ test. Two-sided *P* values were computed and adjusted by the Benjamini-Hochberg method. OR, odds ratio. (**F**) Enrichment analysis between cluster-specific markers and regulons. Hypergeometric *P* value was computed. Only the relationships with Benjamini-Hochberg–adjusted *P* < 0.05 with FE ≥ 2 and the number of overlapping genes ≥ 5 are shown. (**G**) Gene expression of enriched TFs corresponding to the regulons shown in **F**. Color-coded TFs or regulons in **F** and **G** are the ones also identified in mouse monocytes. Red, upregulated; blue, downregulated after trauma.

Because there were no clear boundaries between dendritic cells (DCs), CD14^+^ monocytes, and CD16^+^ monocytes in the UMAP plot (Figure 8B), we analyzed these cell populations separately to deconvolute the circulating monocytes at a higher resolution (scheme shown in Figure 9A). This yielded 14 clusters of myeloid cells that included 1 DCs (cluster 8), 1 CD16^+^ (cluster 4), 1 cycling myeloid cells (cluster 13), 1 monocyte-platelet aggregates (cluster 9), 3 CD14^int^ monocytes (clusters 6, 10, and 12) and 7 CD14^+^ monocytes (clusters 0, 1, 2, 3, 5, 7, and 11). The CD14^+^ monocyte population displayed the most striking differences across time (Figure 9, B–D). To order the clusters in a more biologically meaningful way, we designated each cluster based on assigned cell cluster number (CC#), major cell subset, and enrichment time point (Figure 9E). If a cluster tended to be distributed evenly along time series (highest OR ≤ 2.5), it was labeled as “pan.” The clusters were ordered first by monocyte developmental orders (CD14^+^→CD14^int^→CD16^+^) (28) and then by time series (4h→24h→72h→control) (Figure 9, F and G).

To prioritize critical TFs, we first computed SCENIC (13) regulons using all myeloid cells. Then, we performed enrichment analysis between regulons and identified top markers for each cluster (Figure 9A). Each cluster displayed distinct enriched regulons. There are roughly 2 blocks of TFs/regulons: one associated with CD14^+^ monocytes at early time points after injury and another associated with CD14^+^ cells in the controls (Figure 9F). The expression of corresponding TFs was largely consistent with the regulon enrichment results (Figure 9G). Because regulon computation is independent of the Seurat integration workflow, this analysis provides independent confirmation for the identified clusters.

### Generation of 6 CD14^+^ monocyte signatures.

**Figure 10:**
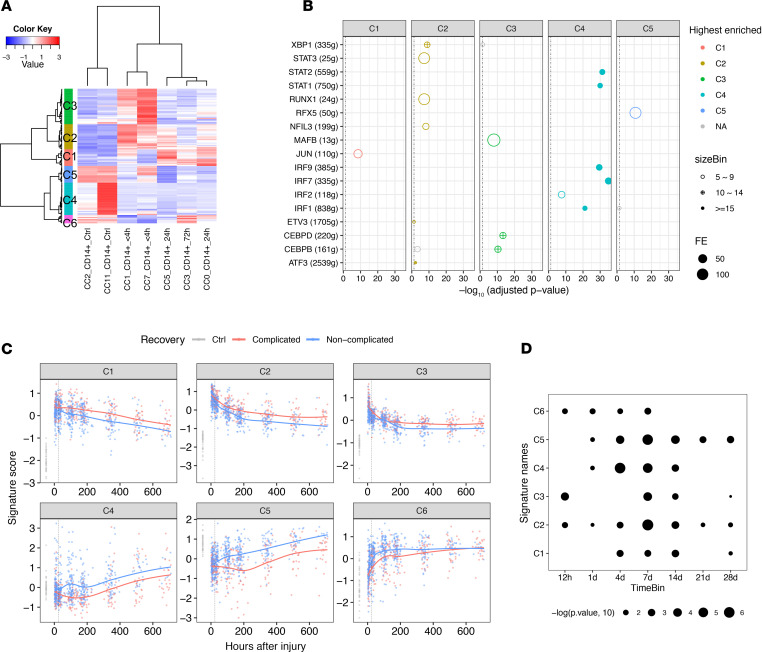
Generation and validation of 6 CD14^+^ monocyte signatures. (**A**) RNA profile of pairwise DEGs (Bonferroni-adjusted *P* < 0.05 and fold change ≥ 2) between 7 CD14^+^ monocyte clusters. Columns represent the average gene expression for each cluster. Genes (rows) are clustered into 6 signatures (C1~C6). Single-cell transcriptomic data were collected from 38 samples harvested at 4 different time points as shown in Figure 8A. (**B**) Enriched regulons for the signatures shown in **A**. Hypergeometric *P* value was computed. Only the relationships with Benjamini-Hochberg–adjusted *P* < 0.05 with FE ≥ 2 and the number of overlapping genes ≥ 5 are shown. Relationships are color-coded by top enriched gene modules (with highest FE). (**C**) Validation of the 6 signatures in published trauma data set (37 healthy controls vs. longitudinal data from 167 patients). Expression of each signature along timeline (up to 28 days after injury) is shown. Smoothing lines were fitted by Loess regression. The vertical dotted line labels the 24-hour time point after injury after injury. (**D**) Statistical quantification of the differences between 2 recovery statuses (complicated vs. noncomplicated) shown in **C** using Wilcoxon’s test. The sampled time points were binned into 7 time points (12 h, 1 d, 4 d, 7 d, 14 d, 21 d, 28 d) after injury. The significant time bin for each signature (Wilcoxon’s *P* < 0.05) is shown.

To further generalize the changes in CD14^+^ monocytes, we identified the pairwise DEGs between each pair of CD14^+^ monocyte clusters. This identified 129 genes above the threshold (adjusted *P* < 0.05 and fold change ≥ 2). These genes could be clustered into 6 signatures in which C1-, C2-, and C3-associated genes were induced after trauma and C4, C5, and C6 represented the features dominant under steady state; these were suppressed after trauma (Figure 10A and Supplemental Table 2). To biologically interpret these signatures, we performed enrichment analysis (Figure 10B and Table 2). C1–C3 were associated with the innate immune response, the response to oxygen-containing compounds, the inflammatory response, and regulation of hematopoiesis. Enriched regulons included *JUN*, *STAT3*, *XBP1*, *MAFB*, *CEBPD*, and *CEBPB*, among others. C4 associated with an IFN-specific program, highly enriched in the regulons of *STAT1*, *STAT2*, and IFN regulatory factors (*IRF1*, *IRF7*, and *IRF9*) (29). It is noteworthy that a small but dominant population of CD14^+^ monocytes (cluster 11, Figure 9F) was highly associated with IFN signaling and significantly enriched in healthy controls. Many C5 genes are MHC II molecules and enriched in the regulon of MHC II activating TF *RFX5* (30). There were only 8 genes in C6, which were not enough to identify significantly enriched GO terms or regulons. Thus, we generalized the changes in monocytes after systemic injury into 6 signatures with central TFs and biological interpretations.

The human and mouse data are generally consistent in the monocyte compartment before and after trauma (Supplemental Figure 9 and color-coded regulons/TFs in Figure 9, F and G). Our findings in human PBMCs further extend the conclusions derived from the mouse T/HS model. Specifically, changes in monocytes do not simply involve a switch between 2 fixed trajectories representing steady state versus posttrauma. Instead, monocytes shift away from steady state in a graded manner (Figure 9C): C1–C3 versus C4–C6 correspond to the 2 distinct directions of the trajectory shifting away or toward steady state.

### Validation of the 6 signatures in bulk RNA data sets.

To provide confirmation for the gene signature patterns derived from our single-cell analysis, we queried a published data set (3) (Supplemental Table 3) of the transcriptomic changes of whole-blood leukocytes from 167 severely injured humans in a 28-day time course, along with a baseline obtained from healthy controls. The changes in the 6 signatures could be largely recapitulated in this database (Figure 10, C and D). Compared with healthy controls, C1–C3 were significantly induced and C4–C6 were suppressed to different degrees after injury. The response to systemic injury was most pronounced at the first sampling time point (~12 hours after injury) and then gradually returned to baseline. The changes were also associated with different clinical trajectories. Complicated cases (time to recovery [TTR] >14 days) showed both higher magnitude and persistence of the changes compared with the noncomplicated cases (TTR ≤ 14 days). It is noteworthy that the changes in 6 monocyte signatures in whole-blood leukocytes may also include the contribution of neutrophils, considering the similar changes between monocytes and neutrophils we observed in mouse BM.

### Two subtypes of trauma patients defined by the 6 signatures with differential prognostic value.

**Figure 11:**
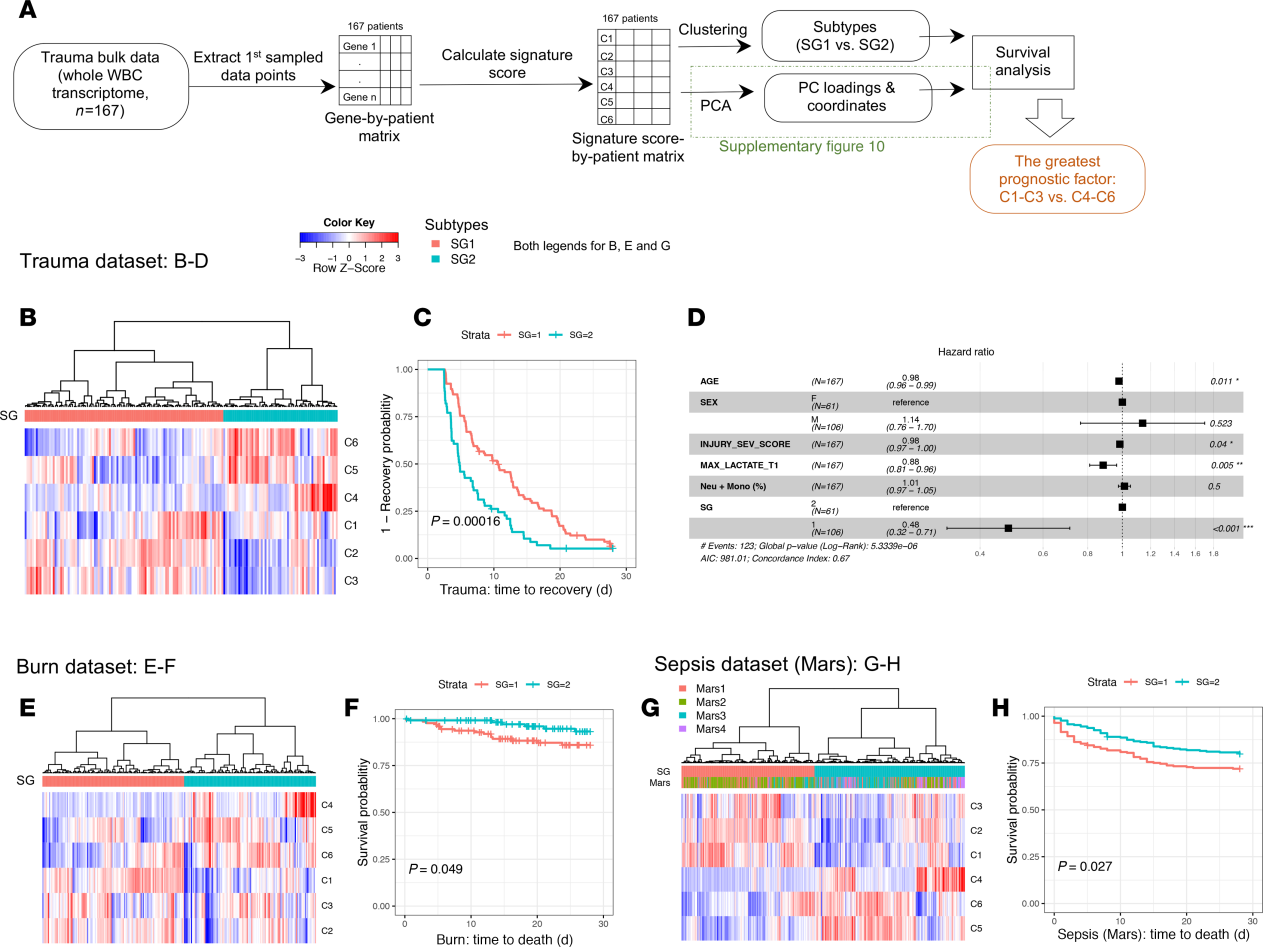
Six signatures define 2 patient subtypes associated with different prognosis. (**A**) Schema describing the workflow for Figure 11 and Supplemental Figure 10. SG, Groups clustered based on Signature scores. (**B**) Trauma patients were clustered into 2 subtypes (SG1 vs. SG2) using the signature score matrix. (**C** and **D**) Time-to-event analyses (event = recovery). (**C**) Kaplan-Meier curve was plotted by the 2 subtypes to visualize 28-day recovery. Log-rank *P* value is shown. (**D**) Hazard ratio of the subtypes after adjusting potential covariates using Cox proportional hazards model. Compared with SG2 (shown as the reference), SG1 is significantly associated with slower recovery after adjusting for the potential covariants. (**E**–**H**) Burn/sepsis patients were clustered into 2 subtypes, and Kaplan-Meier curve was plotted to visualize 28-day survival. Log-rank *P* value is shown. (**B**–**D**) Trauma data set (*n* = 167). (**E** and **F**) Burn data set (*n* = 241). (**G** and **H**) Sepsis data set (*n* = 479).

To explore potential patient heterogeneity in the 6-signature response, we clustered the patients based on a signature score matrix of the 6 signatures (Figure 11A). Because early identification of patients at risk for adverse outcomes could be clinically useful, we extracted the first sampled time point from all 167 trauma patients (mean ± standard deviation: 8.0 ± 3.4 hours). Trauma patients exhibited obvious heterogeneity at the early time point. The 6 signatures roughly clustered the patients into 2 subtypes (SG1 vs. SG2, Figure 11B). Compared with SG2, SG1 patients expressed higher C1–C3 and lower C4–C6 and experienced worse clinical outcomes, including (Table 3) longer hospital length of stay, more severe multiorgan dysfunction, and higher incidence of infectious and noninfectious complications. Kaplan-Meier analysis demonstrated that SG1 patients underwent significantly slower 28-day recovery than SG2 patients (Figure 11C). Well-established prognostic factors for trauma include injury severity (31), brain injury (32), and serum lactate levels (33). To determine the influence of these factors, we assessed patient baseline characteristics (Table 3). Whereas many parameters were distributed evenly, ISS and maximal serum lactate within 6 hours after admission were not. Considering the counts of myeloid cells from this data set may not come from the same sample sent for microarray, we also deconvoluted myeloid composition using RNA data matrix by CIBERSORT (34). Even though SG1 patients had higher ISS and lactate levels within 6 hours, multivariate analysis using a Cox model indicated that SG1 remained an independent risk factor for slower recovery (Figure 11D) after adjusting for these potential covariants, including myeloid composition, approximately 12 hours after injury. These results suggest that patients have intrinsically different responses to systemic injury. Our findings provide additional information for differential prognosis that cannot be explained by injury severity or other known prognostic factors.

In addition to blunt trauma, burns and sepsis are common clinical problems that lead to acute critical illness. To determine if burns and sepsis result in the emergence of similar leukocyte gene expression patterns, we examined burn and sepsis data sets (Supplemental Table 3). We analyzed the first sampled time point after hospitalization (burn) or intensive care unit (sepsis) admission. Similar to the trauma data set, the burn and sepsis patients also fell into 2 subtypes. SG1 patients showed higher C1–C3 and lower C4–C6 signature scores and worse 28-day survival than those in SG2 (Figure 11, E–H). Considering there are 2^6^ combinations of the 6 signatures (up vs. down), we performed PCA on the 6-signature score matrix to comprehensively evaluate the prognostic values of the 6 signatures (Supplemental Figure 10). The sum of the first 3 PCs could explain more than 80% of the variation. Across these diseases, the PCs corresponding to the degree of separation between C1–C3 versus C4–C6 among patients (first PC in trauma and sepsis and second PC in burns) demonstrated the highest and the most significant association with prognosis (*P* values shown in Supplemental Figure 10C).

**Figure 12:**
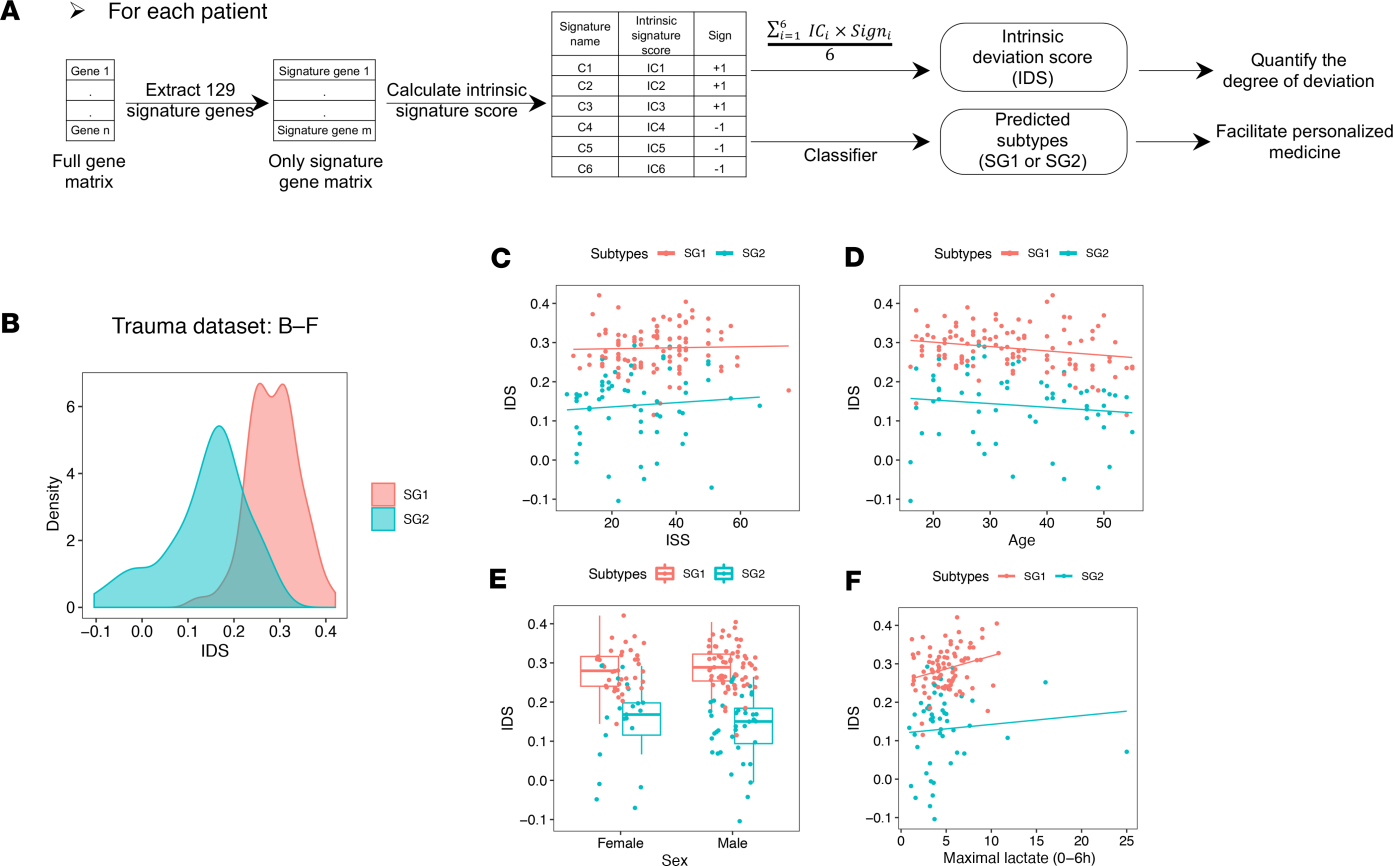
Generation of IDS to give a potential biological explanation for the patient heterogeneity with critical illness. (**A**) Schema describing the workflow for Figure 12 and Figure 13. (**B**–**F**) Two subtypes of trauma patients (*n* = 167) have different IDS distribution (**B**), which cannot be explained by different (**C**) injury severity score (ISS), (**D**) age, (**E**) sex, and (**F**) maximal lactate levels within 6 hours after admission. Data points are color-coded by 2 subtypes. The boxes span from the Q1 to the Q3, with the center line showing the median. Lower whiskers represent Q1 – 1.5*IQR, and upper whiskers represent Q3 + 1.5*IQR.

At the single-cell level, trauma induced an increase in C1–C3 and simultaneous decrease in C4–C6 in CD14^+^ monocytes (Figure 10A). However, we have only discussed the relative expression of each signature among patients (see Methods for the description of the calculation of signature scores). To fill this gap between single-cell observations and patient subtypes, we next determined the relative changes in the genes comprising signatures C1–C6 in individual patients. To quantify this, an “intrinsic signature score” was calculated for each signature. We then generalized the 6 intrinsic signature scores from C1–C6 into a single score, the “intrinsic deviation score” (IDS), to roughly reflect the degree that C1–C3 exceeds C4–C6 in each patient (Figure 12A). SG1 patients displayed a much higher IDS than SG2 patients and this difference could not be explained by age, sex, injury severity or early lactate levels (Figure 12, B–F). The IDS was highly and linearly correlated with the PCs representing the separation based on the expression levels of C1–C3 versus C4–C6 in the population (Supplemental Figure 11). Thus, the changes in CD14^+^ monocytes we characterized at single-cell level may reflect an underlying biological process that results in patient heterogeneity. Because C1–C3 aligned with proinflammatory gene programs and suppressed C4–C6 related to impaired immune responses, it is possible that the excessive and sustained over- “deviation” in these myeloid gene sets contributes to worse prognosis.

### Generation and validation of a classifier for subtype designation.

We have demonstrated that the SG subtypes were consistently associated with different outcomes. Thus, assignment of patients to SG1 or SG2 early after admission could assist with clinical decision-making. Considering SG subtypes were defined based on patient populations, we sought a strategy to translate the observation in individual patients. Thus, we sought a reliable, internal, patient-specific normalization strategy to predict patient SG classification in the future. Considering the distinct distribution of IDS between SG1 and SG2, we built a random forest classifier using the first time point from the 167 published blunt trauma patients (3), taking the 6 intrinsic signature scores for each patient to predict subtype assignment (Figure 12A). After 5-fold crossvalidation we obtained a test error of 0.114 ± 0.046 and AUC = 0.954, indicating that we found an optimal internal normalization to provide a practical way to predict patient SG classification.

**Figure 13:**
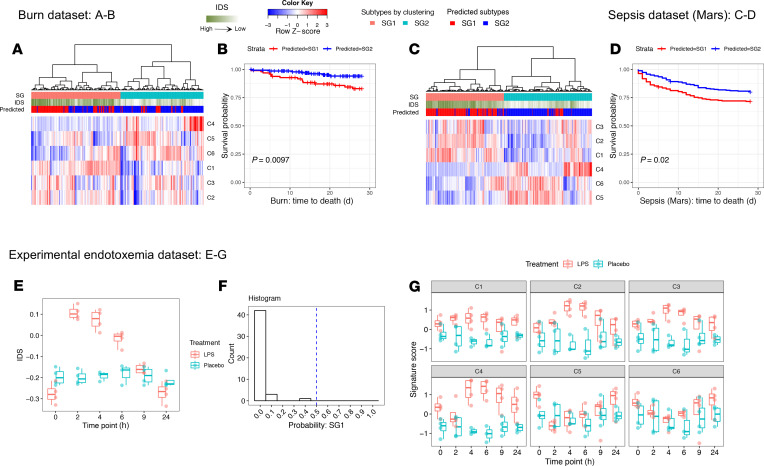
Generation and validation of the classifier for SG subtype designation. (**A**–**D**) For burn/sepsis patients, predicted subtypes and calculated IDS are added to Figure 11, E and G. Kaplan-Meier curve was plotted to visualize 28-day survival between predicted subtypes. Log-rank *P* value is shown. (**A** and **B**) Burn data set (*n* = 241). (**C** and **D**) Sepsis data set (*n* = 479). (**E**–**G**) Endotoxemia data set (LPS: *n* = 4, placebo: *n* = 4). (**E**) Visualization of IDS between 2 groups within 24 hours after LPS or placebo administration. (**F**) Histogram of the predicted probabilities of SG1 in all the data points shown in **E**. (**G**) Expression of the 6 signatures in healthy volunteers within 24 hours after administration of LPS or placebo. The boxes span from the Q1 to the Q3, with the center line showing the median. Lower whiskers represent Q1 – 1.5*IQR, and upper whiskers represent Q3 + 1.5*IQR.

We applied this classifier to human burn, sepsis (35), and experimental endotoxemia data sets (36). The predicted SG1 burn and sepsis patients were consistently associated with worse survival (Figure 13, A–D). Experimental endotoxemia in human volunteers has been used to induce a transient systemic inflammatory response (recovery within 24 hours). Experimental subjects treated with either placebo or endotoxin displayed lower IDS than trauma patients and were all assigned to SG2 (Figure 13, E and F). Experimental endotoxemia induced C1–C3 gene signatures but only minimally suppressed C5 and C6 in circulating leukocytes over 24 hours. Instead of suppression, endotoxin promoted C4 expression (antiviral program) (Figure 13G), which was consistent with previous publications (37, 38). Our analyses highlight the shared and distinct features between a systemic inflammatory response that rapidly resolves (endotoxemia) and one that does not (sustained critical illness) at the single-cell level.

## Discussion

The primary goal of this work was to describe the landscape of transcriptomic changes in circulating immune cells induced by severe injury. Complementary findings in injured mice and humans showed dramatic changes in circulating Ly6C^+^/CD14^+^ monocytes that resulted from the changes in BM. In mice, the trauma-induced changes in monocytes are traceable to progenitor cells in the BM and characterized by 3 simultaneous features, including (a) induction of features of inflammation, (b) suppressed steady-state features, and (c) upregulation of some neutrophil-associated genes. Neutrophils showed similar changes but smaller in magnitude. Our observations in injured humans showed that the monocyte changes can be generalized into 6 signatures with distinct and biologically relevant regulatory networks/TFs. These 6 signatures diverged after injury and further defined 2 patient subtypes associated with different prognosis after severe injury.

The degree of overlap in the inflammatory response between humans and mice has been a topic of debate (39, 40). Some of the lack of consistency between the species is likely due to genetic differences; however, the different composition of cell populations may also be a factor (41). In this study, we independently analyzed and compared the monocyte responses between injured humans and mice in the early phase of the response. The DEGs revealed an intermediate degree of correlation, suggesting consistencies are generally overlapping in the response of the monocyte compartment between the species early after injury. More importantly, many critical TFs and signaling pathways were shared, indicating that the major biological processes are preserved within this cell population.

Two pathways (G vs. M) of monocyte development derived from progenitors have been documented by Yanez et al. (42). The new trajectory derived from BM we characterized in mice can be generally mapped to G pathway (Supplemental Figure 12). In humans, we further demonstrated that the gene expression pattern of monocytes deviates from steady state in a continuous manner after injury, rather than a simple binary pattern. We generalized the degree of deviation into a score (IDS). Experimental endotoxemia induces a lower deviation compared with trauma. More importantly, severely injured patients also exhibit distinct magnitudes of deviation, with thresholds represented in 2 subtypes that cannot be explained by injury severity alone. Thus, by characterizing the full range of transcriptomic patterns observable in monocytes after major systemic perturbations in vivo, our studies go beyond the current model for monocyte development.

We provide evidence that the monocyte gene expression patterns that appear after trauma are also observable in other common acute immunological insults leading to critical illness, including burns and sepsis. A very recent report also identified major changes in CD14^+^ monocytes in patients suffering bacterial urinary tract infections, consistent with the finding that the activation of inflammatory and suppression of MHC II programs in this cell population is a generalizable feature of the early response to trauma and infection (43). Similar features have also recently been reported in COVID-19 patients (44). Thus, the transcriptomic features of immune response we identified within myeloid cells may be a pattern common in critical illness due to many etiologies.

Several transcriptional or clinical subclassifications have been documented for sepsis, including 2 transcriptional subtype analyses (SRS1–2, ref. 45; and Mars1–4, ref. 35) and 1 clinical classification (phenotypes α, β, γ, and δ, ref. 46). Transcriptional subtypes SRS1 and Mars1 have the worst prognosis in the original reports; however, more than 60%–70% of SRS1 patients were mapped to Mars2 rather than Mars1 (Figure S7D in ref. 35). Our subtype SG1 (high deviation and worse prognosis) largely fits with SRS1, Mars2, and clinical phenotype δ. Mars3, Mars4, and SRS2 map to SG2 (low deviation and better prognosis), with Mars4 similar to endotoxemia-like response (Supplemental Figure 13A and Figure 13G). The majority of Mars1 has an intermediate deviation (Supplemental Figure 13, B and C). We noticed that the Mars1-specific PC (PC3, Supplemental Figure 10) was also a prognostic PC achieving statistical significance, suggesting that the biological explanation for the worse outcome in the Mars1 patients is distinct from SRS1 patients. In the future, all of these separate phenotyping efforts may be usefully combined to achieve a more accurate stratification for precision medicine. We advance that goal with our single-cell analyses by linking outcomes in critical illness to specific changes in gene expression in a subset of myeloid cells.

A limitation of our study is the focus of our single-cell studies on the first 3 days. However, we confirmed that gene expression patterns we identified early persisted in patients and further defined 2 subtypes of trauma patients (identifiable as early as approximately 12 hours after injury) with differential prognosis, which were also recapitulated in burn and sepsis patients. We also limited our evaluation of neutrophils to the BM compartment in mice. Further studies will be required to confirm these gene expression patterns persist in circulating neutrophils.

In summary, our landscape findings provide a potentially new paradigm for the immune response to trauma. In the near term, the 2 subtypes of trauma patients could be translated quickly for early identification of the patients at high risk (SG1). In the long term, our findings point to studies on the regulatory mechanisms in mPs and CD14^+^ monocytes as a fruitful area for further research on the mechanisms leading to immune dysfunction after severe injury. Our landscape analysis will act as a new starting point for further study of the regulatory mechanisms and identify the potential target for precision medicine in trauma, which may also be beneficial for other causes of critical illness.

## Methods

Further information can be found in Supplemental Methods.

### Mouse polytrauma model.

We used a previously described mouse model of polytrauma that combines features commonly observed in critically ill trauma patients, including severe hemorrhagic shock and tissue trauma (6, 7). Briefly, anesthetized 8- to 12-week-old male C57BL/6 mice (The Jackson Laboratory, 000664) were subjected to bilateral lower extremity crush injury combined with injection of bone homogenate (a surrogate for long bone fracture). This was immediately followed by hemorrhagic shock for 1.5 hours at a mean arterial pressure of 28–32 mmHg and then resuscitation with lactated Ringers solution at 3 times the volume of the shed blood. We harvested the blood and BM samples at 3 different time points: 3 hours (escalation phase), 6 hours (peak systemic inflammation), and 24 hours (recovery phase) postinjury. Uninjured male littermates were used as controls. Peripheral blood was collected by cardiac puncture for PBMC isolation. Tibias and femurs were collected for BMMC isolation. Young male mice were used for this study because the greatest percentage of severely injured trauma patients are young males (47).

### Patient and human volunteer enrollment.

Patients suffering blunt or penetrating trauma that were admitted to the intensive care unit and experiencing hypotension (systolic blood pressure < 90 mmHg) or tachycardia (heart rate > 108) on admission were eligible for enrollment. Ten patients covering a wide range of age and injury severity were selected for analysis (Supplemental Table 1). Blood samples for PBMC isolation were obtained within 4 hours of injury and at 24 hours and 72 hours after injury. Blood drawn from a healthy age- and sex-matched uninjured volunteer was used to establish the baseline for each patient. The 72-hour samples from 2 patients are not available (1 early death and 1 subject refused the final blood draw), for a total of 38 samples.

### Single-cell cDNA library preparation and sequencing.

PBMCs and BMMCs were isolated by standard Ficoll centrifugation. Single-cell 3′ cDNA libraries were prepared following 10x Genomics protocol (48) (mouse: v2; human: v3). Cases with corresponding controls were processed in parallel within the same batch. Libraries were paired-end sequenced on an Illumina HiSeq platform, with a read length of 150 bp at each end. On average 180 million reads were sequenced for each sample.

### Single-cell sequencing data processing (mouse).

To minimize the potential batch effect, we analyzed each batch of mouse data separately. For each batch, raw sequencing data were processed using the 10x Genomics CellRanger pipeline, *cellranger count* followed by *cellranger aggr* (mouse: v2.1.0, mapped to mm10) to generate a UMI count matrix and then further processed using the Seurat (v2.3.4) (49). Genes expressed in at least 3 cells were retained. Cells with gene counts less than 200 or more than 5000 were filtered out. The number of detected molecules per cell, an unwanted source of variation, were regressed out by the ScaleData() function. PCA was performed on variable genes. Significant PCs were defined by a clear elbow in the plot of the PC standard deviation. Based on the significant PCs, t-SNE, UMAP, and clustering analyses were performed. The resolution for graph-based clustering was tuned back and forth until the identified clusters were biologically meaningful. DEGs between clusters were computed by using the FindMarkers() function with default methods based on the Wilcoxon rank sum test. For specific cell populations of interest, we extracted the UMI count submatrix and redid the secondary analysis mentioned above in order to analyze the differences at a higher resolution. By this analytic workflow, we demonstrated that (a) duplicates largely overlap and (b) the major conclusions can be easily confirmed using different batches.

### Single-cell sequencing data processing (human).

Based on the mouse experiments, biological replicates prepared in parallel were highly reproducible. Thus, similar to the mouse analysis, different time points sampled from the same patients with the matched healthy control were processed by *cellranger count*/*aggr* (v3.0.0, mapped to GRCh38) and then by Seurat (v3.0.2) for quality control and preprocessing in order to largely preserve the differences along timeline. Genes expressed in at least 3 cells were retained. Cells with gene counts less than 200 or more than 5000 or at least 20% mitochondria genes were filtered out. To overcome human heterogeneity and to identify the same cell type or functional state in population, the data from different individuals were integrated by Seurat integration standard workflow (50). To clarify, the integrated data were only used for dimension reduction (e.g., PCA, UMAP, t-SNE) and the downstream analysis taking the results of dimension reduction as input (e.g., clustering). Other analyses were performed based on the original data matrix. For example, DEGs were identified by logistic regression using uncorrected and log-normalized expression data with batch as a potential variable, then corrected by Bonferroni’s method for multiple testing (default method by Seurat).

### Antibodies for flow cytometry.

Fluorophore-conjugated antibodies against myeloid lineage markers (CD11b, Ly6G, Ly6C, CD115), a TF IRF8 with IgG1 κ isotype control, other lineage markers (CD3ε, TCRγδ, B220, NK1.1, Ter119, CD19), and leukocyte common antigen CD45 were listed as below: LIVE/DEAD Fixable Aqua Dead Cell Stain Kit (Invitrogen, Thermo Fisher Scientific, catalog L34965), anti-CD3ε FITC (145-2C11) (eBioscience, Thermo Fisher Scientific, catalog 11-0031-85), anti-TCRγδ FITC (GL3) (eBioscience, Thermo Fisher Scientific, catalog 11-5711-82), anti-B220 FITC (RA3-6B2) (eBioscience, Thermo Fisher Scientific, catalog 11-0452-82), anti-NK1.1 FITC (PK136) (BD Biosciences, catalog 553164), anti-Ter119 FITC (TER-119) (BioLegend, catalog 116205), anti-CD19 FITC (1D3) (BD Biosciences, catalog 553785), anti-CD45 BUV395 (30-F11) (BD Biosciences, catalog 564279), anti-CD11b PE-Cy7 (M1/70) (BioLegend, catalog 101216), anti-Ly6G APC-Cy7 (1A8) (BD Biosciences, catalog 560600), anti-Ly6C PerCP-Cy5.5 (HK1.4) (eBioscience, Thermo Fisher Scientific, catalog 45-5932-82), anti-CD115 PE (AFS98) (eBioscience, Thermo Fisher Scientific, catalog 12-1152-81), anti-IRF8 APC (V3GYWCH) (eBioscience, Thermo Fisher Scientific, catalog 17-9852-80), and anti-IgG1 κ isotype control APC (P3.6.2.8.1) (eBioscience, Thermo Fisher Scientific, catalog 17-4714-82).

### Computation of PC-associated genes and PC functional annotation.

For the PC of interest, we computed Pearson’s correlation between the scaled expression value by Seurat (49) and PC coordinates for each gene. A Benjamini-Hochberg–adjusted *P* ≤ 0.05 was used as the cutoff to define PC-associated genes. With the correlation coefficient as the rank, GSEA was performed using the fgsea R package (v1.6.0). The top enriched gene sets with positive normalized enrichment scores largely indicated the biological functions on the PC positive side and vice versa.

### Pseudotime estimation.

Pseudotime was computed by the Monocle (19) R package (v2.8.0) using the default parameters taking UMI matrix as input. To avoid the influence of cell cycle phases, pseudotime was computed after removing all cell cycle genes based on GO term annotation (GO: 0007049). We forced the mP-enriched state as pseudotime 0. Among the variable genes identified from Seurat (49), the genes differentially expressed along pseudotime were identified by the differential GeneTest() function. The genes with *q* < 0.001 (*q* value was provided by Monocle) were used to build up the transcriptomic profile and cluster into gene modules.

### Regulon detection.

Myeloid regulons were computed with the SCENIC (13) R package (v1.0.1.1) using the UMI count matrix of myeloid cells with the default parameters. The computed regulons were further used as the gene sets for enrichment analysis or to calculate signature scores.

### Enrichment analysis.

For enrichment between 2 gene sets, for a preranked gene list, GSEA (12) was performed using the fgsea R package (v1.6.0); for the gene lists without rank, “a” represents the number of shared genes between gene set 1 and gene set 2; “b” represents the number of genes only in gene set 2; and “c” represents the number of genes only in gene set 1. Universe genes (N) were defined as the genes expressed in at least 0.5% of the cells used to compute corresponding gene modules. FE was computed as below:

  (Equation 1).
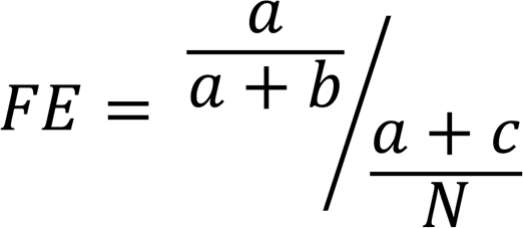


The hypergeometric *P* value for enrichment was computed and adjusted by the Benjamini-Hochberg method for multiple testing.

For enrichment or deletion between human monocyte clusters and time points, the 2-sided *P* values of the χ^2^ test and OR were computed. The *P* value was adjusted by the Benjamini-Hochberg method.

### Signature score calculation.

Signature scores were calculated as the average expression of the signature genes (or probes for microarray data) after *z* score transformation across the patients, as described by Guo et al (51). The “signature score matrix” based on the 6 human monocyte signatures was calculated in this way.

### Intrinsic signature score and IDS calculation.

We defined intrinsic signature scores (annotated as IC1–IC6, corresponding to the 6 signatures C1–C6) in order to reflect relative expression of the 6 signatures within an individual. For each patient, 6 intrinsic signature scores were calculated as follows: (a) extraction of the log_2_ transformed expression values of 129 signature genes from the full transcriptomic data; (b) *z* score transformation across all signature genes; and (c) for each signature, the corresponding *z* score–transformed values were averaged to get the intrinsic signature score. Thus, each patient was assigned 6 values.

Random forest was used to establish the classifier, taking the 6 intrinsic signature scores as input to predict which subtype the patient should belong to. We used all the first sampled data points from 167 trauma patients as the training data set with 5-fold crossvalidation. Subtypes were obtained from clustering analysis. Burn and sepsis data sets with available survival data were used as 2 independent validation cohorts.

C1–C3 were the signatures induced after trauma, so their signs were “+1.” C4–C6 were the signatures suppressed after trauma, so their signs were “–1.” IDS was calculated by the equation shown below:

  (Equation 2).
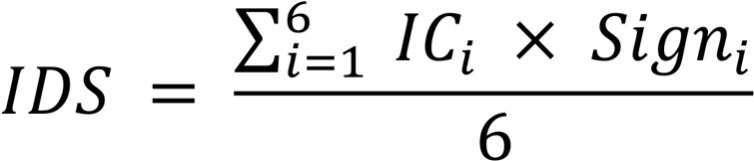


### Time-to-event analysis.

For the gene array data sets that have multiple time points for each patient (trauma and burn), only the first sampled gene array data points were included for the survival analysis. For trauma patients, event was set as recovery status because of the very few cases of in-hospital deaths (160 alive vs. 7 dead, mortality rate: 4.2%). The definition of recovery was based on the annotation from the original data set (3). For nonsurvivors, “hospital length of stay” was used as the time, and recovery status was annotated as “No.” For burn and sepsis patients, the event was set as in-hospital death. A Kaplan-Meier curve was plotted by survival R package (v2.43.3) for visualization of 28-day prognosis and the log-rank *P* value was computed. Cox proportional hazards model was performed by coxph() function in R adjusting for covariates, including age, sex, serum lactate within 6 hours, and severity (if available).

### Data and materials availability.

The raw scRNA-Seq data sets (both human and mouse) in the FASTQ format with filtered gene/barcode matrix have been uploaded to the National Center for Biotechnology Information’s Gene Expression Omnibus (GSE162806).

### Statistics.

Our analyses ranged from circulation to BM, from mouse to human, from single-cell transcriptome to whole-blood leukocyte transcriptome, from trauma to other critical illness (sepsis and burn). The major changes have been validated by at least 2 independent data sets. The ggplot2 R package (version 3.0.0) was used for customized data visualization.

For DEG identification from scRNA-Seq data, mouse DEGs were computed using Wilcoxon’s rank sum test. Due to the potential influence of batch effect in human analyses, human DEGs were identified by logistic regression using uncorrected and log-normalized expression data with batch as a potential variable. The detected DEGs were adjusted by Bonferroni’s method (default method by Seurat) for multiple testing. Adjusted *P* < 0.05 was considered significant.

### Study approval.

Mouse experimental protocols were approved by the Institutional Animal Use and Care Committee of the University of Pittsburgh. Experimental procedures were carried out in accordance with all regulations regarding the care and use of experimental animals (National Institutes of Health).

Trauma patients and healthy volunteers were enrolled in an observational study approved by the University of Pittsburgh Institutional Review Board. Written informed consent was obtained from all the subjects (or next of kin).

## Author contributions

TRB and TC designed the study. TC performed data analysis. WC supervised the statistical methods. KC supervised single-cell cDNA library preparation and sequencing. TC set up the cell isolation system in the mouse model. AJL set up the cell isolation system in the human samples. JLS and RAN supervised clinical data, cell collection, and annotation. JC prepared single-cell cDNA libraries. TC and PL performed flow cytometry. TRB, YV, and MD supervised the data interpretation in trauma. LLM, PAE, and MJD supervised the interpretation of the human clinical data from *The Inflammation and Host Response to Injury* data sets and the data on MDSC signatures. DCA and CS supervised the data interpretation in sepsis. TRB, TC, LLM, and MJD wrote the manuscript with the feedback of KC, JLS, RAN, AJL, MD, JC, PAE, PL, CS, DCA, YV, and WC, who have read and approved the manuscript.

## Supplementary Material

Supplemental data

## Figures and Tables

**Table 1:**
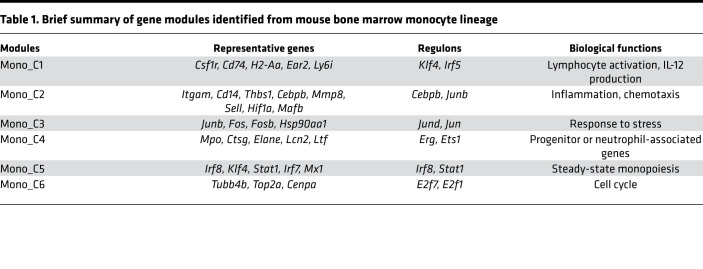
Brief summary of gene modules identified from mouse bone marrow monocyte lineage

**Table 2:**
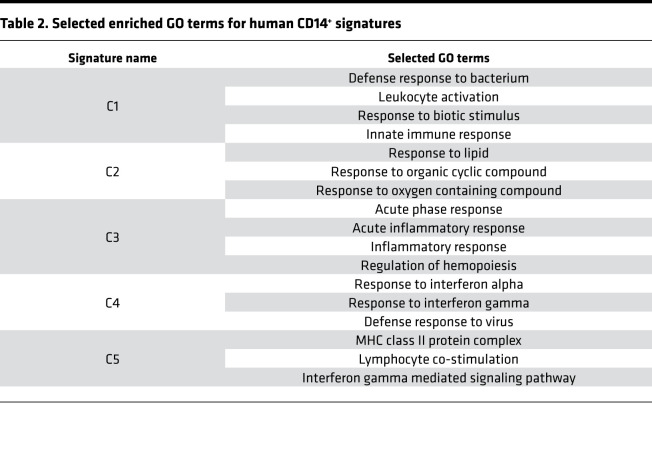
Selected enriched GO terms for human CD14^+^ signatures

**Table 3:**
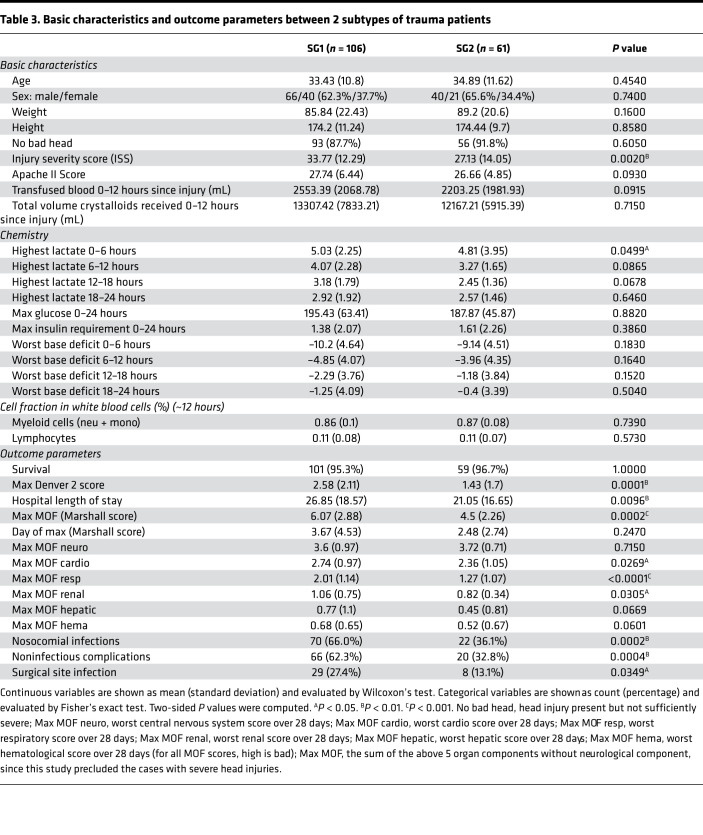
Basic characteristics and outcome parameters between 2 subtypes of trauma patients

## Version 1 12/15/2020

In-Press Preview

## Version 2 01/25/2021

Electronic publication
