# Supplementary material for: A road map from single-cell transcriptome to patient classification for the immune response to trauma
Source: JCI Insight. 2021 Jan 25;6(2):e145108. doi: 10.1172/jci.insight.145108 (PMC7934885; doi:10.1172/jci.insight.145108)
Supplement: Supplemental data [file jciinsight-6-145108-s129.pdf]

Supplemental Materials:

PBMC and BMMC isolation

Mouse PBMC were isolated by standard procedure of Ficoll gradient centrifugation (Ficoll-Paque Premium 1.084, Cat#17-5446-02, GE Healthcare). Residual red blood cells were removed using red blood cell lysing buffer (R7757, Sigma-Aldrich). Bone marrow was obtained from tibias and femurs by flushing the marrow cavities with 3ml PBS (containing 2% FBS and 2mM EDTA). BMMC were isolated by Ficoll gradient centrifugation using the same protocol described for mouse PBMC. Human PBMC were isolated by Ficoll-Paque PLUS (Cat#17-1440-03, GE Healthcare) using SepMate tubes (Stemcell) to accelerate the procedure. Other steps were largely the same as the mouse protocol.

The isolated cells were cryopreserved and thawed for analysis according to the 10x Genomics protocol (1). Briefly, PBMC were resuspended in 0.5ml resuspension medium (40% FBS in DMEM) and 0.5ml freezing medium (40% FBS + 30% DMSO in DMEM) in a 1:1 ratio. BMMC were resuspended in 1ml freezing media (90% FBS + 10% DMSO). Cells were chilled in CoolCell (Corning) in -80 overnight and transferred to liquid nitrogen. Cryopreserved vials were thawed in the water batch at 37°C, removed from water bath when a tiny ice crystal remained and then transferred to a 50ml conical tube after thawing was complete. A milliliter of thawing medium (PBMC: 10% FBS in DMEM; BMMC: 20% FBS in PBS) was added dropwise (5 sec/drop), followed by 2ml, 4ml, 8ml, 16ml thawing medium at ~ 1-min intervals. After this, the cells were washed and resuspended in calcium and magnesium free buffer (PBMC: PBS with 0.04% BSA; BMMC: PBS with 10% FBS) for cDNA library preparation.

Cell staining for flow cytometry

Fresh mouse BMMC isolated by Ficoll centrifugation from control and T/HS (6hr) were stained for flow cytometry, 2 mice/groups. Transcription Factor Staining Buffer Set from eBioscience was used for

intracellular staining of IRF8. Experiment was repeated for three times. Analysis was performed on by flow cytometer (LSR-15) and analyzed with FlowJo software. The $CD45^+$ live cells in the monocyte lineage were defined by 2 gating strategies: (1) $Lin^-CD115^+Ly6G^-$ and (2) $Lin^-Ly6C^{++}Ly6G^-$. Multi-dimensional protein data of $Lin^-Ly6C^{++}Ly6G^-$ gated monocytes were also visualized by Matlab/Cyt3 (2). The compensated data (fcs files) of gated populations were taken as the input and Arcsinh transformed. The cells from each mouse were down-sampled to 3000 cells for visualization.

Doublet and low-quality cell removal

Different cell types have different number of expressed genes. Eg. monocytes have more expressed genes compared with lymphocytes. One hard cutoff was not be applicable for all cell types. Thus, in initial quality control, we used a relatively low threshold to include more potential high-quality cells. In the analysis of a specific population, doublets and low-quality cells were more distinguishable at the higher resolution, usually forming small isolated clusters. Doublets were identified based on the biological knowledge, e.g. co-expressing both T and B cell markers. Low-quality cell clusters were identified by: (1) Most top genes were still mitochondria genes after initial quality control or (2) The number of expressed genes was extremely lower than other clusters and in the absence of cluster-specific genes. Thus, doublet and low-quality cell removal was an iterative process.

Regulon detection and PCA projection

For motif references (from https://resources.aertslab.org/cistarget/), "mm10__refseq-r80__500bp_up_and_100bp_down_tss.mc9nr.feather" and "mm10__refseq-r80__10kb_up_and_down_tss.mc9nr.feather" RcisTarget databases were downloaded for mouse regulon detection. "hg38__refseq-r80__10kb_up_and_down_tss.mc9nr.feather" and "hg38__refseq-r80__500bp_up_and_100bp_down_tss.mc9nr.feather" were downloaded for human regulon detection.

Regulons were projected onto PCA 2D space largely following the published methods (3, 4). Each single cell had a coordinate for each PC and was assigned an AUC (area under curve) value for each regulon by SCENIC. Pearson's correlation was calculated between AUC values and PC coordinates. Finally, each regulon was plotted on PCA 2D space based on the correlation coefficients with corresponding two PCs, respectively.

Cell cycle phase assignment

Cell cycle phases were computed by the cyclone() function from scran R package (v1.8.2). The UMI count matrix was taken as the input. The pre-defined classifiers provided with the scran package were used to assign cell cycle phases.

RNA velocity computation

RNA velocity of myeloid progenitors was computed by velocyto (5) (v0.17). The mouse specific reference, "mm10_rmsk.gtf", was downloaded from UCSC genome browser as expressed repeats annotation. "genes.gtf" was generated by *cellranger mkref* as a genome annotation file. The *cellranger count* output was taken as the input to generate a loom file for each sample. Loom files from different samples were merged using the loompy python package. Finally, RNA velocity was estimated using the velocyto R package (v0.6).

Hierarchical clustering

Ward clustering was performed using hclust() function in R with the agglomeration method set as "ward.D2" (6). The distance matrix was 1 minus Pearson's correlation. Hierarchical clustering was used to identify gene or patient clusters.

Generation of customized signatures

(1) Circulating monocyte markers (Supplemental Figure 2B): To roughly estimate the developmental relationship among mouse circulating monocytes, GSE95702 (7) was used to generate this signature. The monocyte development referenced in this paper is MDP (monocyte-macrophage DC progenitors) → cMoP (common monocyte progenitors) → BM Ly6C^{+} → blood Ly6C^{+} → blood Ly6C^{int} → blood Ly6C^{low}. We identified the genes that were up-regulated in circulating monocytes compared with the monocyte progenitors and Ly6C^{+} monocytes in BM. DEGs were computed using the limma R package (v3.36.3) on the log2 transformed expression values with Benjamini-Hochberg adjusted p-value = 10^{-4} as the cutoff.

(2) Monocyte differentiation associated genes (Supplemental Figure 4B): To validate the pseudotime of the monocyte lineage, we extracted the genes which are positively or negatively correlated with monocyte differentiation in bone marrow from the published dataset GSE95702 (7). First, PCA was performed on the samples of MDP, cMop and BM Ly6C^{+} monocytes. PC1 largely represented the differentiation process of monocytes in bone marrow, with the above 3 populations aligning from the left, middle and to the right. For each gene, we computed Pearson's correlation between the log2 transformed expression value and PC1 coordinates. Benjamini-Hochberg adjusted p-value ≤ 0.05 was used as the cutoff to define the genes which are significantly associated with PC1. Among these genes, the ones with positive Pearson's correlation coefficient are the genes which are gradually down regulated along monocyte differentiation (signature name: Diff down), and those with negative Pearson's correlation coefficient were gradually up regulated along monocyte differentiation (signature name: Diff up).

(3) MDSC signatures (Supplemental Figure 7A): To evaluate immunosuppressive potential, signatures for MDSC from 5 different sources were extracted from the published dataset GSE21927 (8). Generally, different sources of MDSC were compared with the CD11b^{+} counterparts isolated from healthy spleen from the same species. DEGs were computed using the limma R package (v3.36.3) on the expression

values (already log2 transformed). The genes that were significantly up-regulated in MDSC were used to identify MDSC signatures. The cutoff was set as Benjamini-Hochberg adjusted p-value = 10^{-3} for BM-derived MDSC and 10^{-5} for tumor-derived MDSC.

Data processing of GEO microarray data

RNA microarray data of inflammatory diseases and platform information tables were downloaded from the GEO database. For each dataset, the workflow is generally as follows:

(1) The expression matrix was log2 transformed for untransformed data.

(2) PCA was performed to evaluate the data quality. The trauma dataset displayed 2 obvious batches. These 2 batches align on the opposite sides of PC1 which represents 51% of the variation as shown in the figure below. We double checked the meta data, all samples from batch 2 were annotated as either the

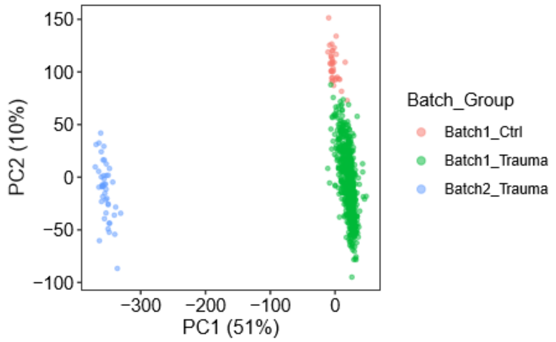

samples with low RNA quality or with incomplete time points. Thus, we excluded batch 2 and only used the batch 1 for validation. The other data sets did not show obvious batch effects.

(3) For each probe, the log2 transformed expression matrix was z-score transformed across all the samples for signature score calculation.

Clinical annotations of trauma dataset

In the trauma dataset, trauma patient outcomes were originally classified as an uncomplicated when time to recovery (TTR) was <5 days, intermediate when TTR was ≥ 5 and ≤ 14 days and complicated when

TTR was >14 days or the patient died within 28 days. TTR was defined as the number of days following the trauma event until organ dysfunction had resolved (9). For the analysis of this data set, we pooled intermediate and uncomplicated cases together and classified the patients into either complicated (TTR >14 days or death) or non-complicated (TTR ≤ 14 days) categories (Figure 10C). All clinical annotations were downloaded from http://www.gluegrant.org/.

Deconvolution of cell composition

To prepare the input of the array data, CEL files were downloaded from GSE36809 and then processed by CEL_to_mixture.R provided by CIBERSORT. For the input of signature matrix, we used the signature matrix of LM22, which is CIBERSORT provided and contains 22 functionally defined human immune subsets. We used the deconvoluted results for the 1st time points of 167 trauma patient and added the neutrophil + monocyte percentage into the cox regression model shown in Figure 11D.

Signature score calculation in details

(1) For mouse single-cell RNA sequencing data, the average of Seurat (v2.3.4) (10) scaled values (stored in @scale.data slot which are normalized, scaled, log and z-score transformed) was the signature score for each cell. (2) For GEO datasets, the expression values in the matrix were log2 transformed (if this had not already been performed) and z-score transformed. Then, the average of the log2 and z-score transformed expression values was defined as the signature score for each subject.

We noticed that MDSC signatures contains multiple cell cycle genes. In this case, cell cycle phases would become a significant cofounder, especially in the BM. Thus, MDSC signatures were calculated after removal of all the cell cycle genes based on the GO term annotation (http://www.informatics.jax.org/go/term/GO:0007049).

Entrez ID – gene symbol and mouse – human homolog gene exchange

Genome wide annotation for mouse (org.Mm.eg.db, v3.6.0) and for human (org.Hs.eg.db, v3.6.0) were installed. Entrez ID – gene symbol exchange was performed by AnnotationDbi R package (v1.42.1). The HomoloGene data file was downloaded from ftp://ftp.ncbi.nlm.nih.gov/pub/HomoloGene/current. Mouse entrez IDs were mapped to human homolog entrez IDs using annotation Tools R package (v1.58.0)

Supplemental Figures:

- PBMC, 6hr (A-E)

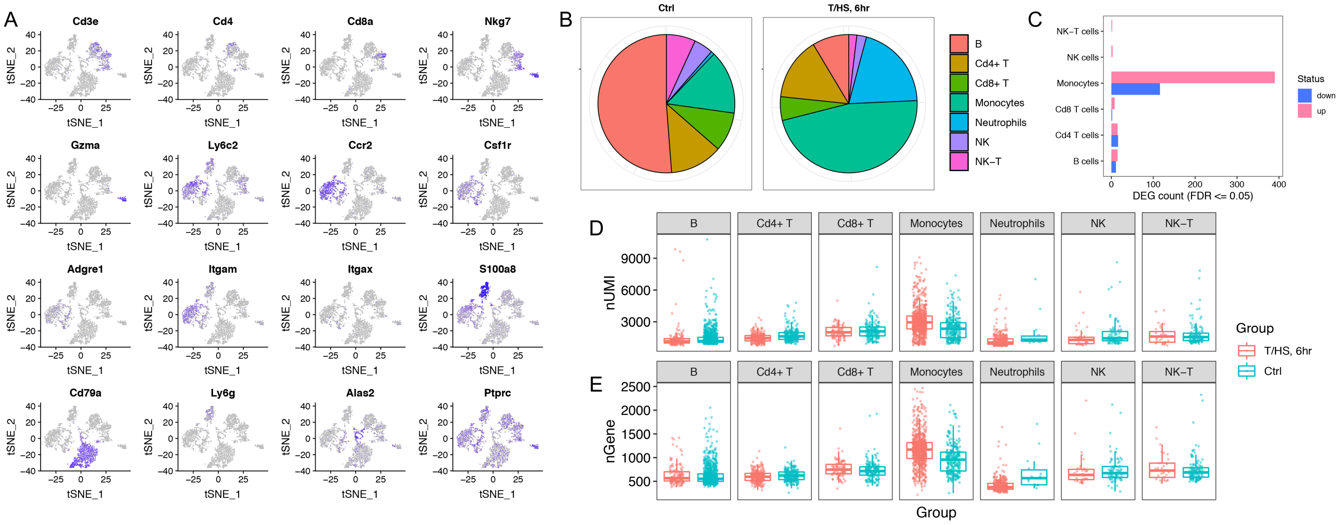

Supplemental Figure 1. Overview of transcriptomic changes of PBMC at 6hrs after T/HS. Related to Figure 1-2. (**A**) t-SNE plot of PBMC as shown in Figure 1B. The expression of representative markers is shown. (**B**) Changes in the fractions of major cell types in PBMC after T/HS. (**C**) The number of DEG detected in each cell type (adjusted p-value < 0.05). (**D-E**) The number of molecules (UMI: unique molecular identifier) (D) and detected genes (E) are shown by cell types + groups. Each dot represents a cell.

A

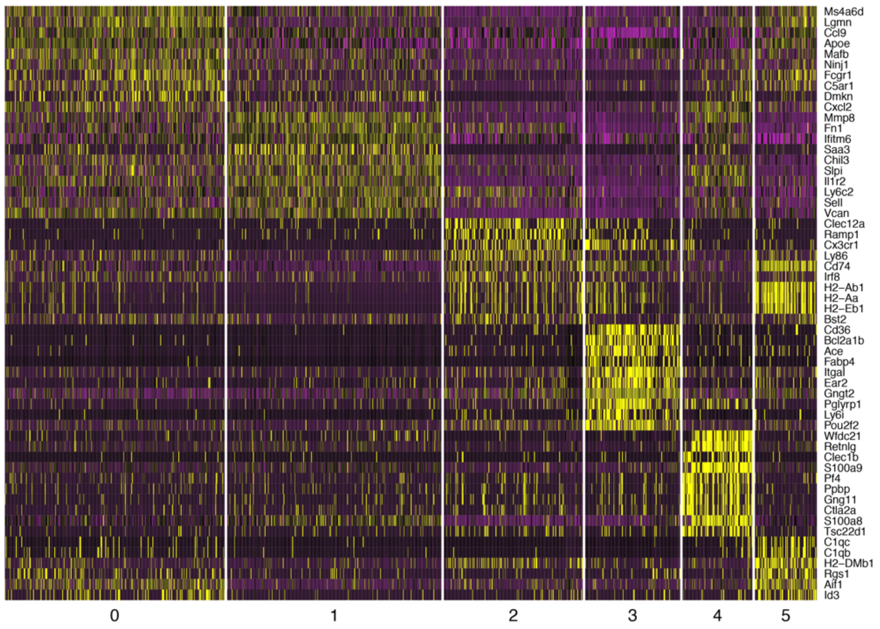

- GSE95702 (B-C)

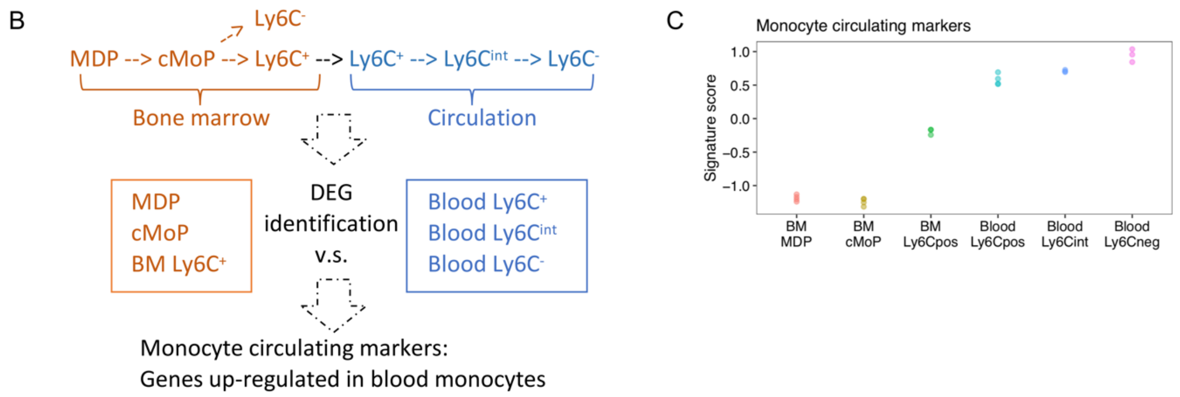

Supplemental Figure 2. Overview of transcriptomic changes of monocytes at 6hrs after T/HS. Related to Figure 1-2. (**A**) Top markers for each monocyte cluster as shown in Figure 1C. Cells (columns) are ordered by clusters. (**B**) Schema describing the workflow for how monocyte circulating markers were extracted from GSE95702 dataset (Supplemental Methods). (**C**) Visualization of the expression of extracted monocyte circulating markers in monocyte subsets characterized in GSE95702 dataset, which confirms the extracted signatures are indeed the genes up-regulated in circulating monocytes.

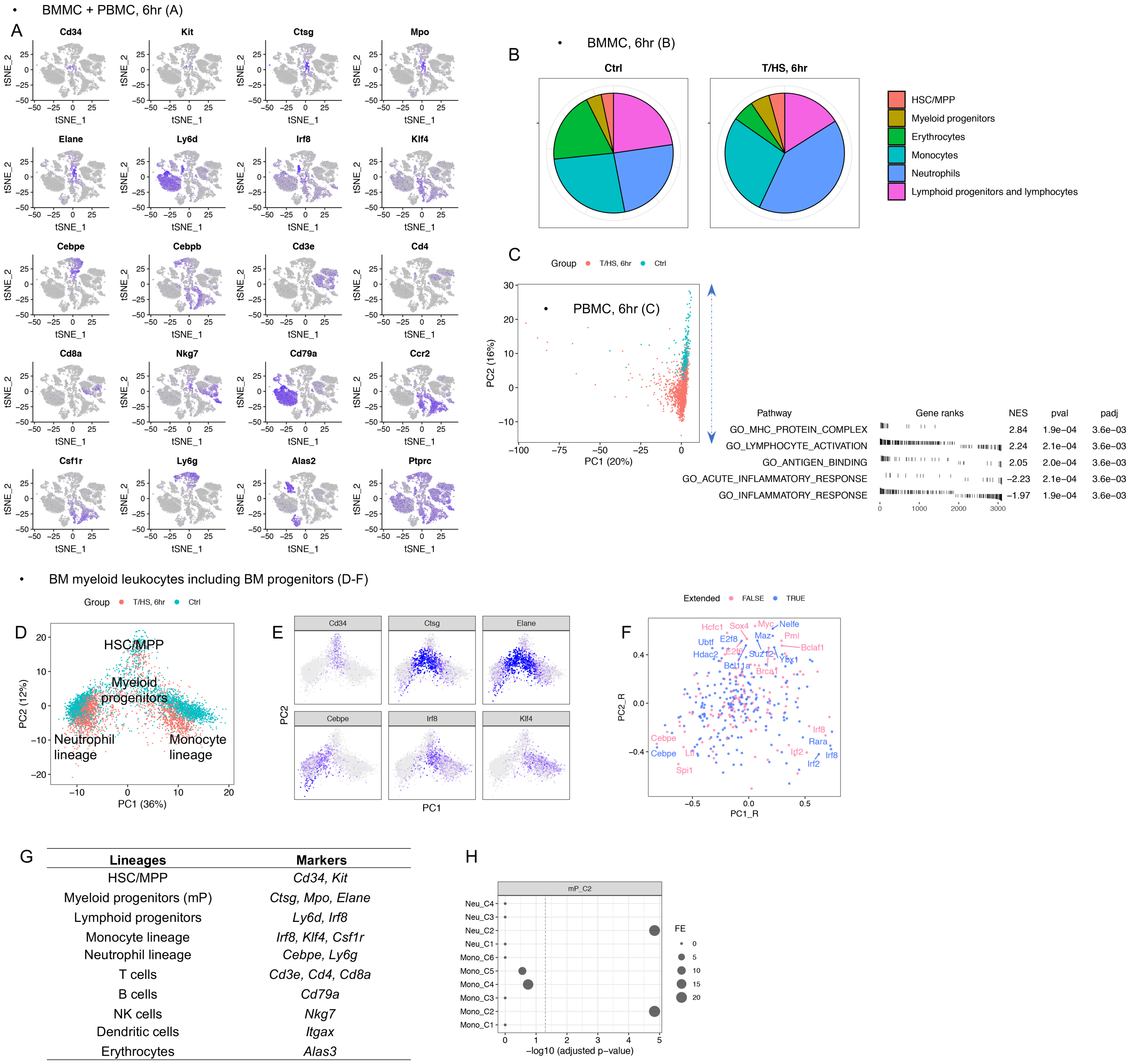

Lineages	Markers
HSC/MPP	*Cd34, Kit*
Myeloid progenitors (mP)	*Ctsg, Mpo, Elane*
Lymphoid progenitors	*Ly6d, Irf8*
Monocyte lineage	*Irf8, Klf4, Csf1r*
Neutrophil lineage	*Cebpe, Ly6g*
T cells	*Cd3e, Cd4, Cd8a*
B cells	*Cd79a*
NK cells	*Nkg7*
Dendritic cells	*Itgax*
Erythrocytes	*Alas3*

Supplemental Figure 3. Overview of transcriptomic changes of paired BMMC + PBMC at 6hrs after T/HS. Related to Figure 3-5. (**A**) t-SNE plot of paired BMMC + PBMC as shown in Figure 3B. Expression of representative markers are shown. (**B**) Changes in the fractions of major cell types in BMMC after T/HS. (**C**) PCA plot shows circulating monocytes from this batch color coded by groups. PC2 largely represents the changes after T/HS. Selected enriched GO terms of PC2-associated genes by

GSEA are shown. Thus, the circulating monocytes from this batch reproduced the major changes from the first experiment as shown in Figure 2A-2B. (**D**) PCA (PC1 vs. PC2) plot of BM myeloid cells, including HSC/MPP + myeloid progenitors, color coded by groups. PC1 vs. PC3 is shown in Figure 4A. Expression of lineage markers are shown in (**E**). (**F**) Projection of the regulons on the PCA 2D space as shown in (D-E) (Supplemental Methods). Well-established TFs (e.g. *Cebpe* ~ neutrophils (11), *Irf8* ~ monocytes (11), *Sox4* ~ stem cells (12)) largely overlay with the known corresponding lineages, supporting the reliability of the computed regulons. (**G**) Lineage markers used to distinguish major cell types or lineages. (**H**) Enrichment analysis is performed between mP_C2 and each gene module identified from the BM monocyte and neutrophil lineages, demonstrating that the features of mP_C2 are preserved in the downstream lineages. Hypergeometric p-value was computed and then adjusted by Benjamini-Hochberg methods. Black vertical dash line annotated where adjusted p-value is equal to 0.05.

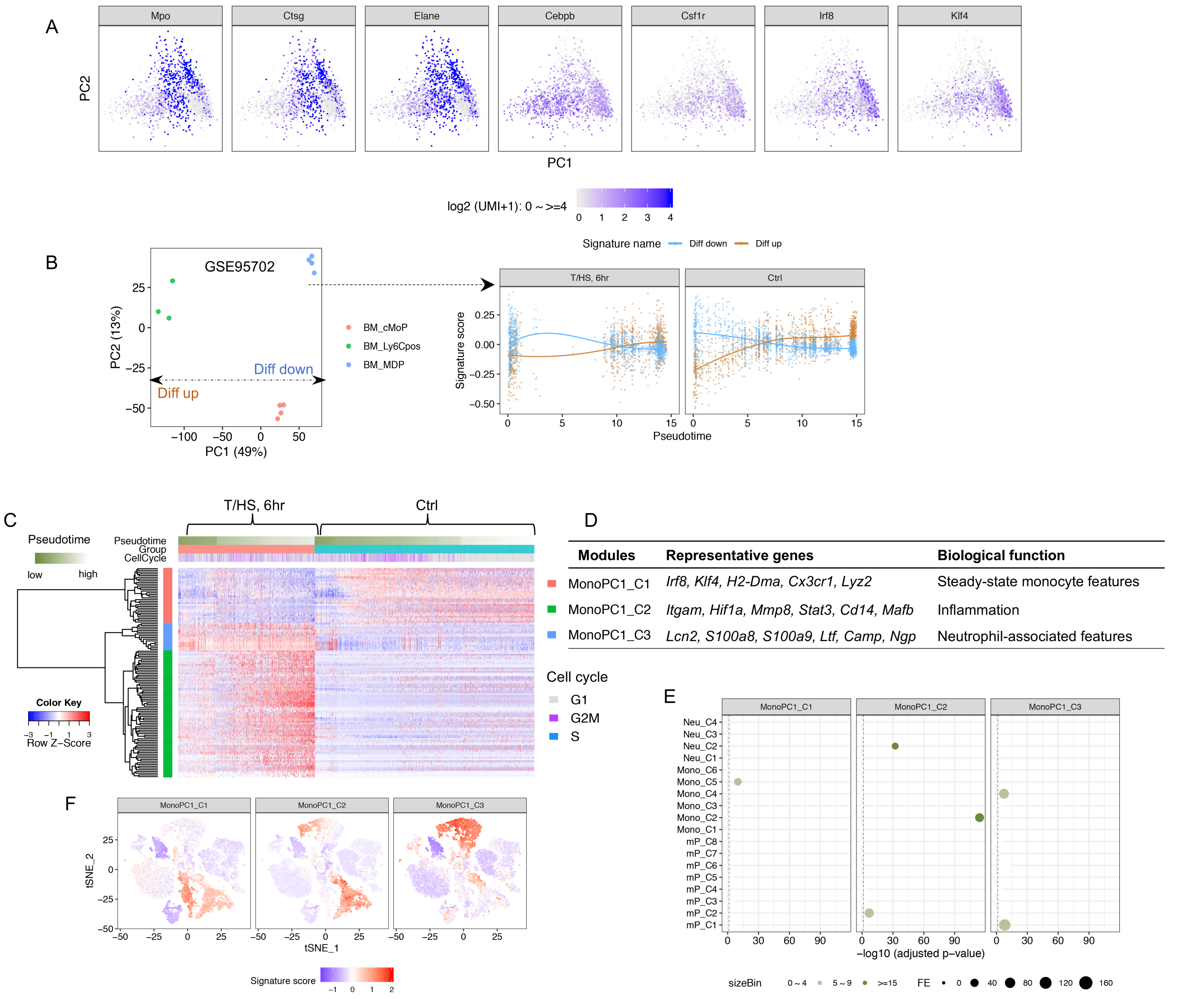

Supplemental Figure 4. Characterization of transcriptomic changes in the BM monocyte lineage at 6 hrs after T/HS. Related to Figure 6-7. (**A**) Expression of representative markers are shown in the PCA plot as Figure 6B. (**B**) To validate the computed pseudotime, we extracted genes positively and negatively associated with BM monocyte differentiation from the GSE95702 dataset (Supplemental Methods). Signature scores were calculated for each single cell shown in PCA plot in Figure 6B and plotted along pseudotime. Smoothing lines were fitted by Loess regression. (**C**) Extraction of the major differences between groups from the PC1 as shown in Figure 6B. Ward clustering yielded three gene modules

(MonoPC1_C1~C3) of PC1-associated genes (Pearson's correlation: adjusted p-value < 0.05 and $|r| \geq 0.3$). (**D**) Summary of the three gene modules identified in (A). (**E**) Enrichment analysis between the three gene modules and mP/monocyte/neutrophil gene modules identified at 6 hrs in mouse BM. Relationships are colored by binned number of overlapping genes. Only the relationship with ≥5 overlapping genes are visible. Mono_C1-C6 were characterized in Figure 6-7, Neu_C1-C4 in Supplemental Figure 6 and mP_C1-C8 in Figure 5. (**F**) Visualization of the signature scores in the t-SNE plots as shown in Figure 3B.

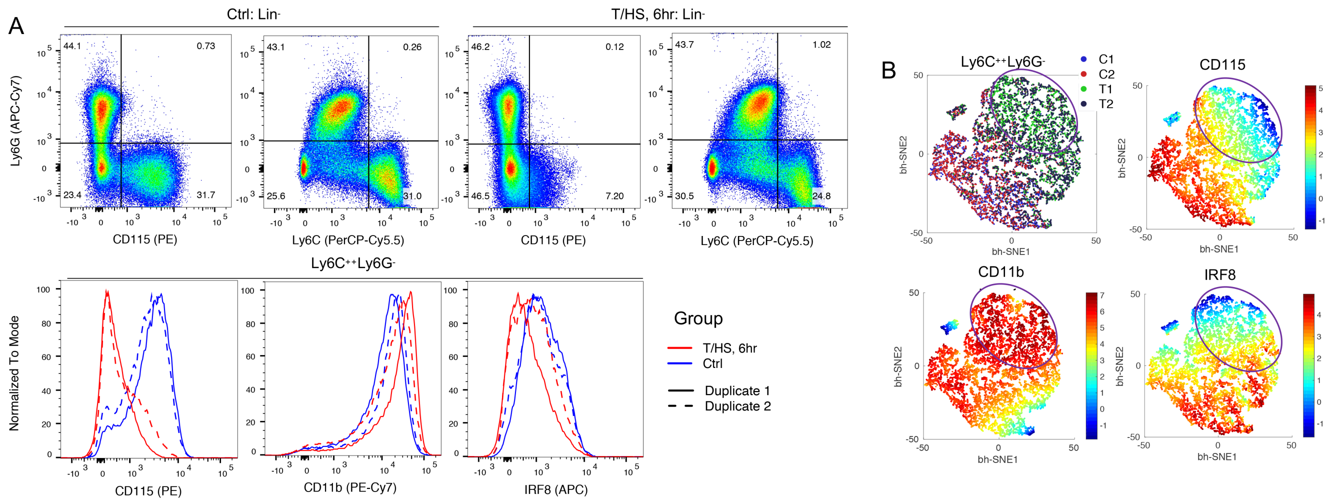

Supplemental Figure 5. Validation of transcriptomic changes in the BM monocyte lineage at 6 hrs after T/HS by flow cytometry. The results from one experiment (2 mice/group) are shown, which has been repeated for two extra times. (**A**) FlowJo analysis. (**B**) Matlab/Cyt3 analysis of $Lin^{-}Ly6C^{++}Ly6G^{-}$ monocytes (C: Ctrl; T: T/HS, 6hr).

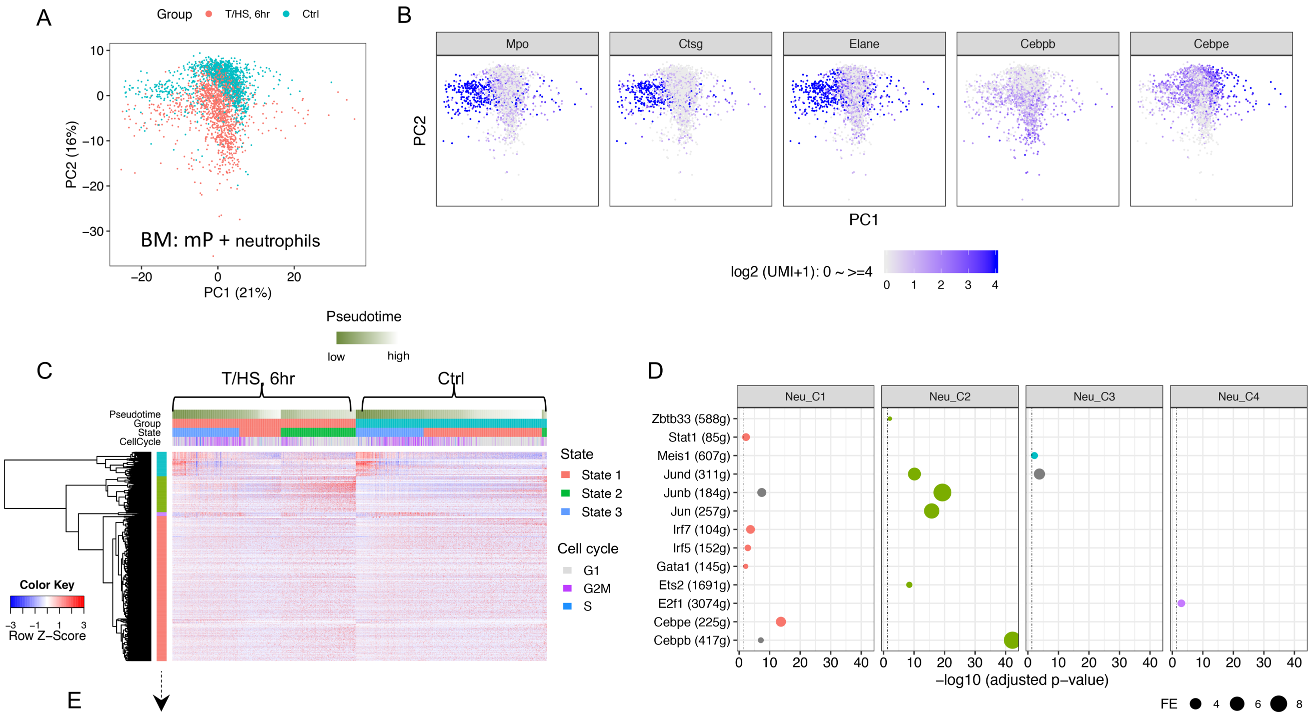

E

Modules	Representative genes	Regulons	Biological functions
Neu_C1	*Ly6g, Cd74, Tnf, H2-Ab1, H2-Aa*	*Stat1, Irf5, Cebpe*	Steady state features
Neu_C2	*Il1b, Cebpb, Mmp8, Thbs1, Socs3*	*Cebpb, Junb, Jund*	inflammation
Neu_C3	*Mpo, Elane, Ctsg, Ramp1, Ncl, C1qbp*	*Meis1*	Progenitor or monocyte associated features
Neu_C4	*Pcna, Mki67, Cenpf, Tubb5*	*E2f1*	Cell cycle, proliferation

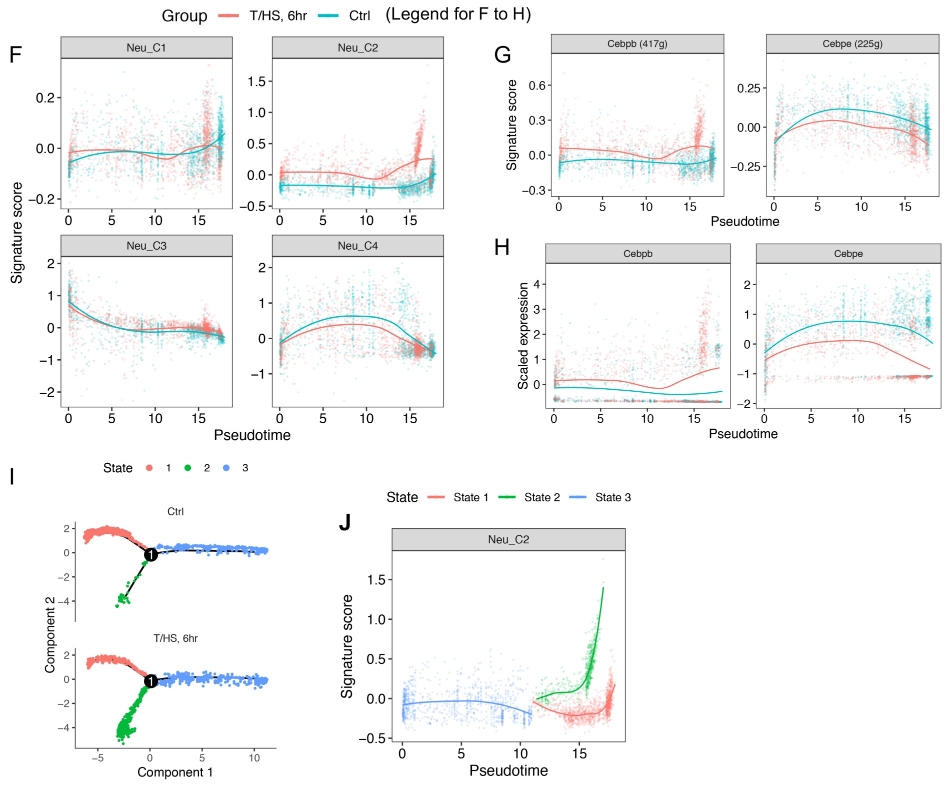

Supplemental Figure 6. Characterization of transcriptomic changes in the BM neutrophil lineage at 6 hrs after T/HS. (**A**) PCA plot of BM neutrophil lineage including all myeloid progenitors as shown in the 4th panel of Figure 3B, and expression of representative markers are shown in (**B**). (**C**) RNA profile of the BM neutrophil lineage is built upon pseudotime associated genes computed by Monocle 2. Cells (columns) are ordered first by groups, then by states identified using Monocle2 and lastly by pseudotime (from progenitors to committed cells). Genes (rows) are clustered by Ward clustering into four gene modules (Neu_C1~C4). (**D**) Enrichment analysis between the four gene modules and regulons. Hypergeometric p-value was computed. Only the relationships with Benjamini-Hochberg adjusted p-value < 0.05 with fold enrichment (FE) ≥ 2 and the number of overlapping genes ≥ 15 are shown. Relationships are color coded by top enriched gene modules (highest FE). (**E**) Summary of the four gene modules. (**F**) Expression of the four gene modules, (**G**) critical regulons and (**H**) corresponding TFs along pseudotime color coded by groups. Smoothing lines were fitted by Loess regression. (**I**) Trajectories constructed by Monocle2 are color coded by states and wrapped by groups. (**J**) Expression of Neu_C2 along pseudotime color coded by states.

A

GSE21927

Strains	MDSC	compared with	Control
BALB/c	CD11b+ cells from bone marrow cultured with GM-CSF+IL-6		CD11b+ cells from healthy spleen
BALB/c	CD11b+ cells from 4T1 tumor bearing mice		CD11b+ cells from healthy spleen
BALB/c	CD11b+ cells from C26-GM tumor bearing mice		CD11b+ cells from healthy spleen
C57BL/6	CD11b+ cells from EL-4 tumor bearing mice		CD11b+ cells from healthy spleen
C57BL/6	CD11b+ cells from MCA203 tumor bearing mice		CD11b+ cells from healthy spleen

- Genes up-regulated in MDSCs were defined as MDSC signatures.
- Total 5 signatures for different sources of MDSCs.

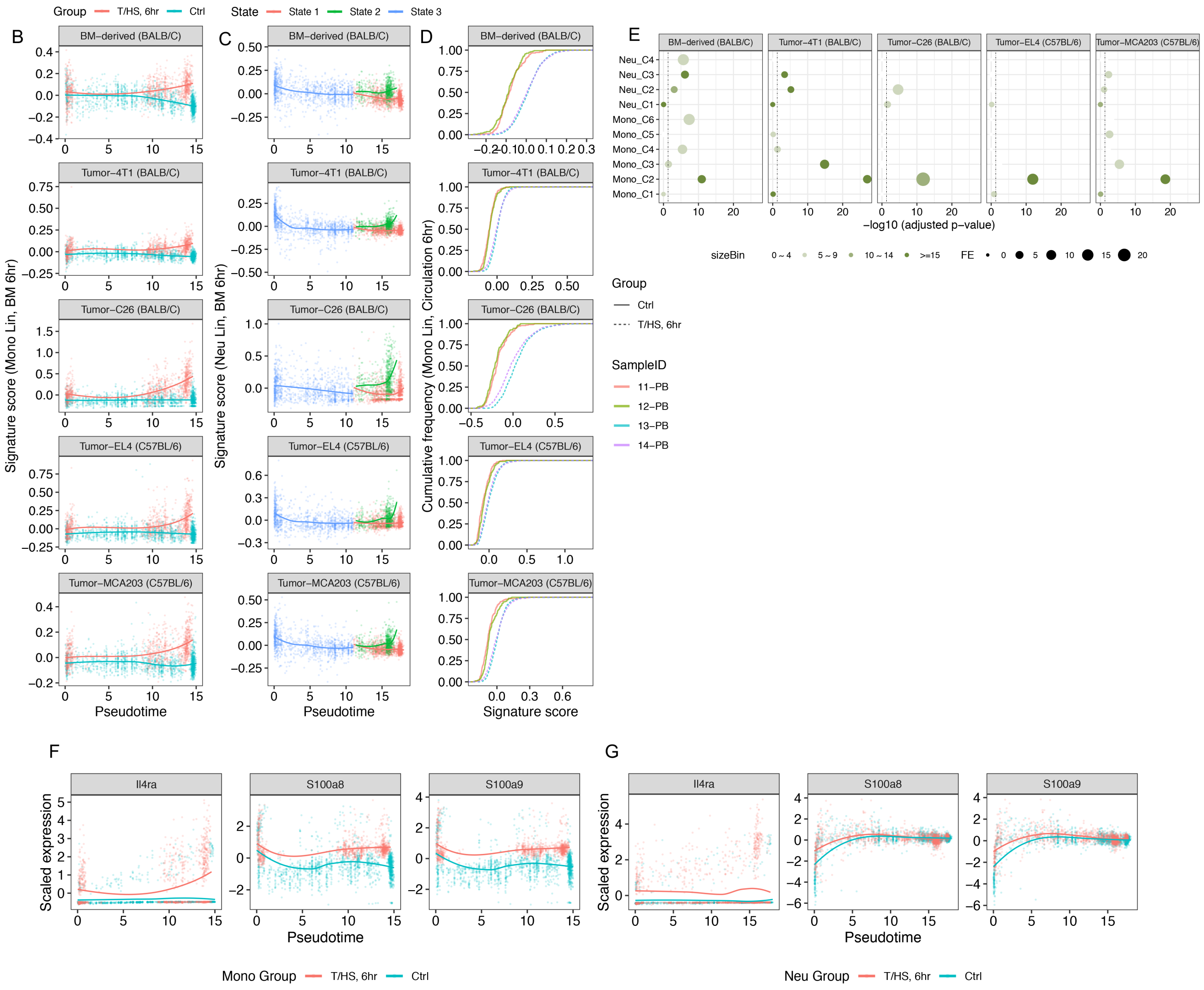

Supplemental Figure 7. Simultaneous initiation of inflammatory and features of myeloid-derived suppressor cells (MDSC) during emergency myelopoiesis. (**A**) Development of MDSC gene signatures (Supplemental Methods). (**B-C**) MDSC signatures are up-regulated in BM monocytes after T/HS (B) and in BM neutrophil stimulated state (state 2) (C) along pseudotime. A smoothing line was fitted by Loess

regression. (**D**) MDSC signatures are up-regulated in circulating monocytes after T/HS. Cumulative frequencies of signature scores (CDF curves) are shown. (**E**) Enrichment analysis between MDSC signatures and each gene module identified from BM monocyte and neutrophil lineages at 6hrs after T/HS. Hypergeometric p-value was computed. Relationships are color coded by binned number of overlapping genes. Only relationships with ≥5 overlapping genes are visible. The MDSC signatures were significantly enriched in the inflammatory modules (Mono_C2 and Neu_C2, *Cebpb* regulon associated), especially Mono_C2. (**F-G**) RNA expression of some functional markers associated with MDSC in BM monocytes (F) and BM neutrophils (G) after T/HS. In general, transcripts for functional markers (13) (*S100a8*, *S100a9*, *Il4ra*) and TFs (13) (↑*Cebpb*, ↑*Stat3*, ↑*Stat5* and ↓*Irf8*) associated with MDSC also increased after T/HS (Figure 4C, 7B-7D, Supplemental Figure 6G-6H). These observations indicate that in addition to pro-inflammatory changes, features associated with MDSC are also initiated in the new trajectory.

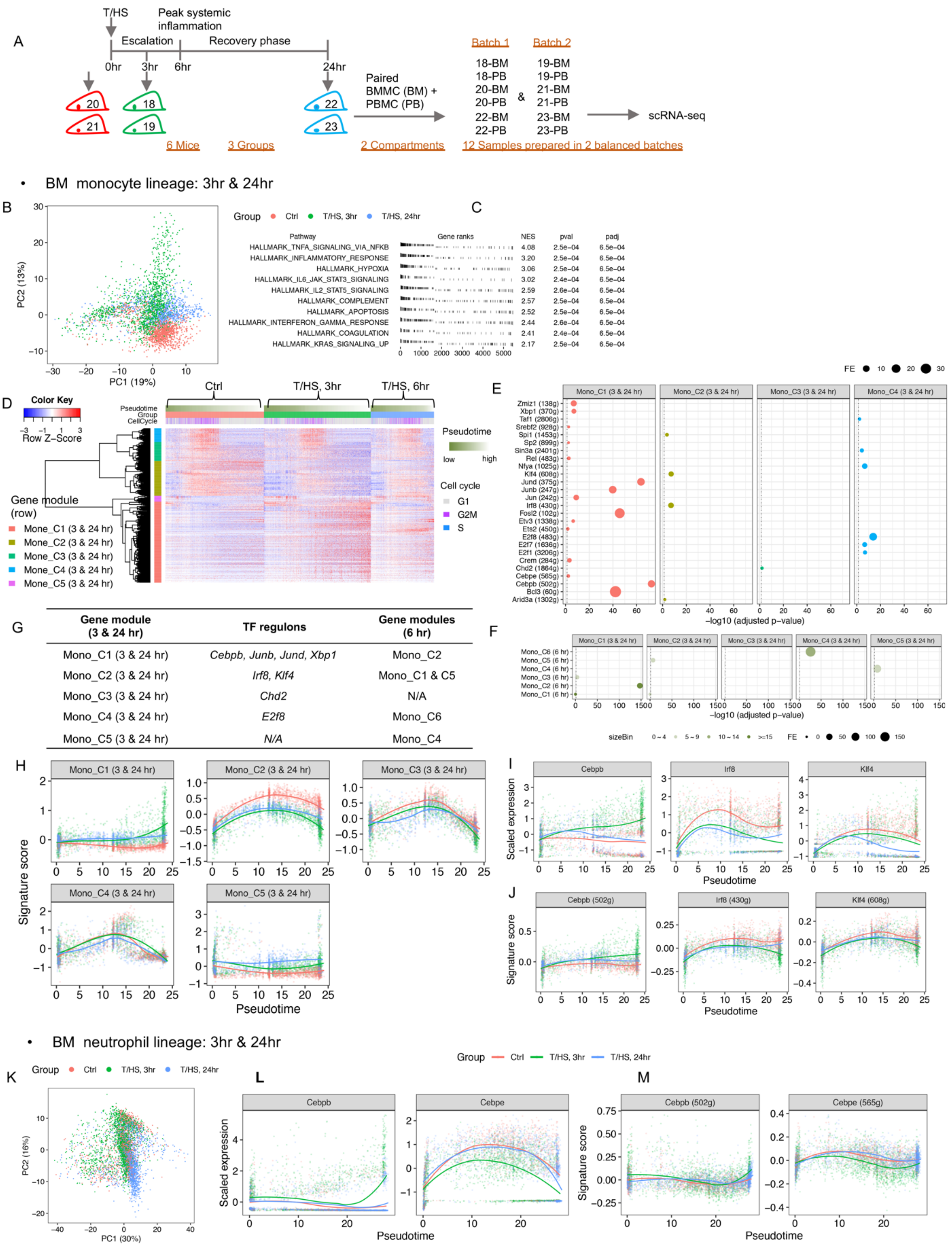

Gene module (3 & 24 hr)	TF regulons	Gene modules (6 hr)
Mono_C1 (3 & 24 hr)	*Cebpb, Junb, Jund, Xbp1*	Mono_C2
Mono_C2 (3 & 24 hr)	*Irf8, Klf4*	Mono_C1 & C5
Mono_C3 (3 & 24 hr)	*Chd2*	N/A
Mono_C4 (3 & 24 hr)	*E2f8*	Mono_C6
Mono_C5 (3 & 24 hr)	*N/A*	Mono_C4

Supplemental Figure 8. Characterization of transcriptomic changes in the BM monocyte and neutrophil lineages at 3 hrs and 24 hrs after T/HS. (**A**) Experimental design of isolating paired BMMC + PBMC at 3 hrs and 24 hrs for scRNA-seq. (**B-J**) Analysis of BM monocyte lineage at 3hrs and 24hrs after T/HS. (B) PCA plot of BM monocyte lineage. The changes induced by T/HS are represented by PC2. (C) Top 10 hallmark pathways enriched in the positive side of PC2 by GSEA. (D) RNA profile was built upon PC2-associated genes (Pearson's correlation: $|r| \geq 0.2$ and adjusted p-value ≤ 0.05). Cells (columns) are ordered first by groups then by pseudotime. Cell cycle stages are also labeled. Genes (rows) are clustered into 5 gene modules, Mono_C1~C5 (3 & 24 hr). (E) Enrichment analysis between identified gene modules and regulons. Regulons were computed based on the myeloid cells including HSC/MPP and myeloid progenitors at 3hrs and 24hrs. Hypergeometric p-value was computed. Only the relationships with adjusted p-value < 0.05 and fold enrichment (FE) ≥ 2 and the number of overlapping genes ≥ 15 are shown. (F) Map the gene modules identified at 3 & 24 hrs to the ones at 6 hrs (enrichment analysis). Relationships are colored by binned number of overlapping genes. (G) The gene modules derived from the 6hr time point were largely recapitulated at 3hrs and 24hrs. (H-J) Expression of each gene module (H), critical TF (I) and corresponding regulon (J) along pseudotime. Smoothing lines were fitted by Loess regression. (**K-M**) Analysis of BM neutrophil lineage at 3hrs and 24hrs after T/HS. (K) PCA plot of BM neutrophil lineage. Although BM neutrophils did not show obvious global transcriptomic changes 3 & 24hrs, the critical TFs (L) and corresponding regulons (M) followed a similar trend to those seen at 6hrs shown in Supplemental Figure 6.

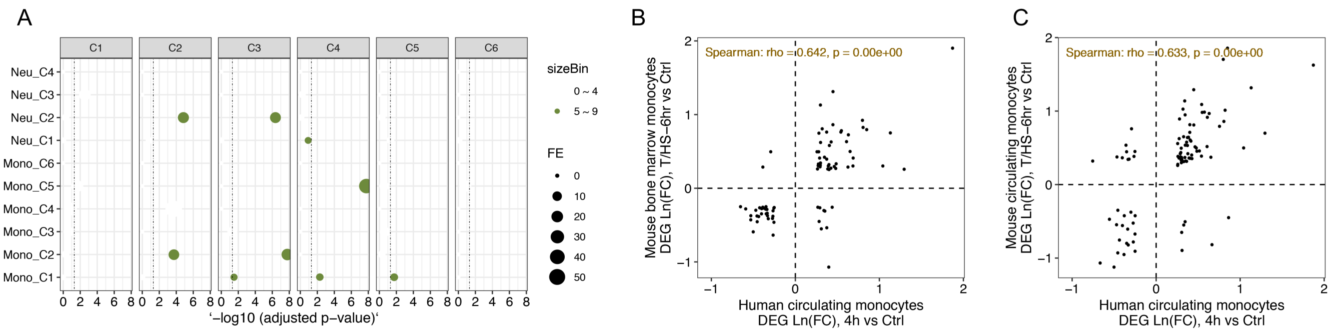

Supplemental Figure 9. Summary of the overlapping features: The human and mouse data are generally consistent in the monocyte compartment before and after trauma. (**A**) Enrichment analysis between six human signatures (C1-C6) and the monocyte/neutrophil modules identified from mouse BM (Mono_C1-C6 & Neu_C1-C4) at 6hrs after T/HS. Hypergeometric p-value was computed and adjusted by Benjamini-Hochberg method. Relationships are colored by binned number of overlapping genes. Only relationships with ≥ 5 overlapping genes are visible. Correlation of monocyte DEG identified from mouse BM vs. human PBMC (**B**) or from mouse PBMC vs. human PBMC (**C**). FC: fold change.

The human and mouse data are generally consistent in the monocyte compartment before and after trauma. The six human monocyte signatures can be mapped to mouse bone marrow gene modules. Human monocyte signatures C2 and C3 overlap with mouse inflammatory modules Neu_C2 and Mono_C2. S100A8 and S100A9, both neutrophil-associated genes, are contained in human C1. Human C4 (IFN signaling) and C5 (MHCII signaling) correspond to mouse steady-state modules Mono_C5 (*Irf8, Irf7*) and Mono_C1 (lymphocyte activation, including CD74 and *H2-Aa*), respectively. Globally, shared DEG derived from human and mouse monocytes after trauma display an intermediate level of correlation (Spearman correlation: $\rho > 0.6$).

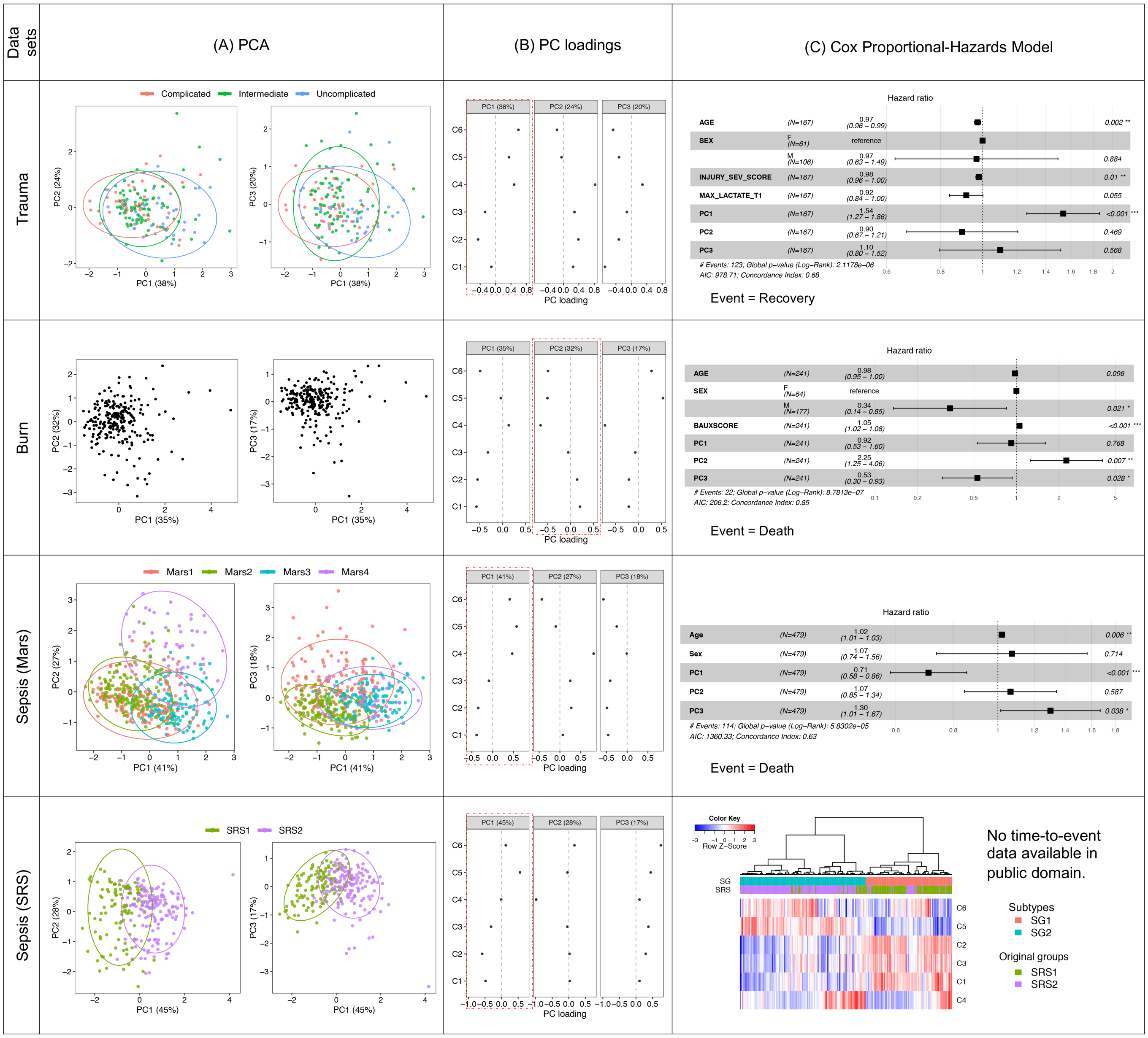

Supplemental Figure 10. Systemic characterization of the prognostic value for the six signatures. Related to Figure 11. (**A**) PCA was performed on the signature score matrix. Patients were color coded by the groups demonstrated in the original paper. Ellipses indicate 95% confidence interval. (**B**) The signature loadings on first three PCs are shown. The PCs representing the degree of separation between C1-C3 vs. C4-C6 among patients are highlighted in red blocks. (**C**) The prognostic values of the first three PCs were evaluated by a Cox model after adjustment of age, sex and severity (if available). The names of datasets

are labeled in the first column (Supplemental Table 3). No groups documented for burn dataset, so all the patients are in black. Sepsis SRS dataset do not provide time-to-event data. Thus, clustering results are shown instead.

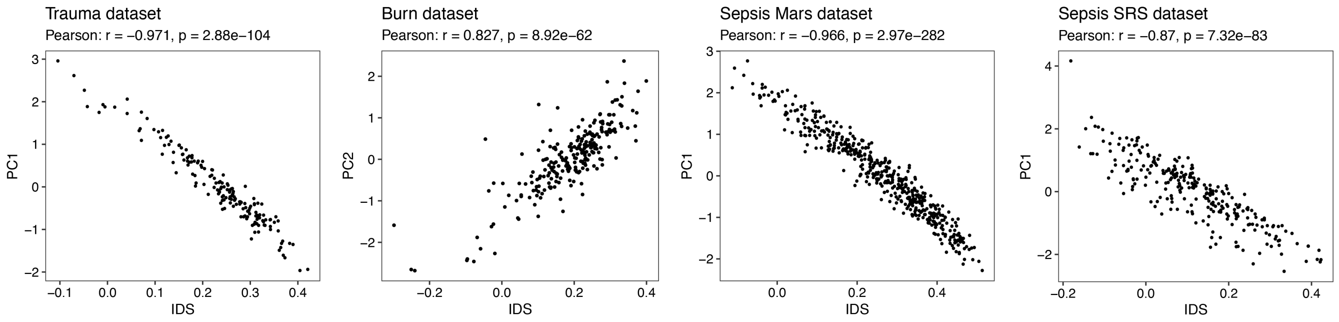

Supplemental Figure 11. Correlation between IDS and the PC highlighted in red blocks shown in Supplemental Figure 10.

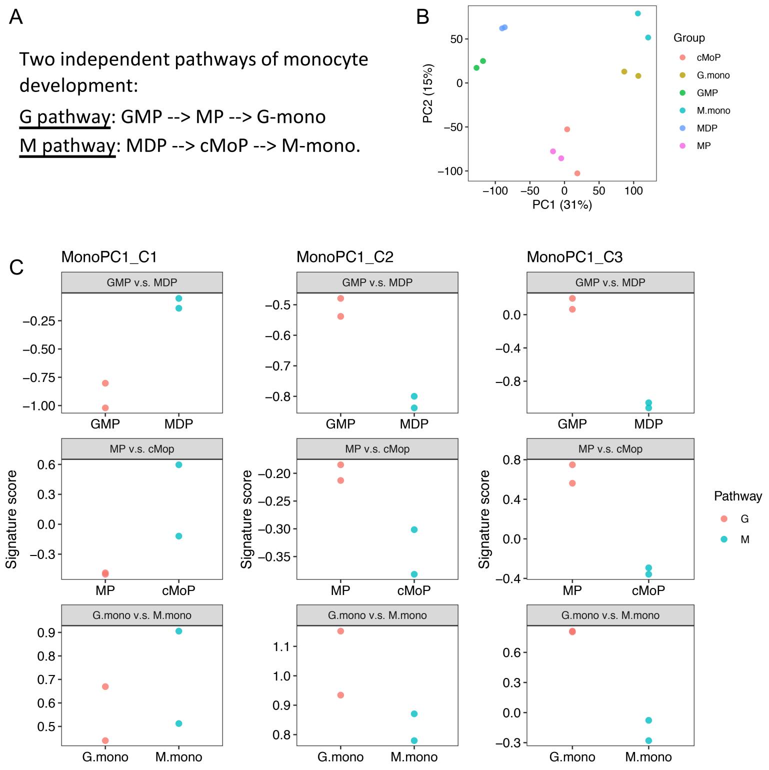

Supplemental Figure 12. Similarity between the new trajectory of monocytes after T/HS and the G pathway characterized by *Yanez et al* (14). (**A**) Introduction of the major points in the paper from *Yanez et al*. (**B**) Gene expression matrix was downloaded from GSE88982 GEO dataset. PCA was performed on the six subsets involved in monocyte development. This is largely consistent with Figure 3A in the original paper. (**C**) Signature scores of MonoPC1_C1-C3 (characterized in Supplemental Figure 4, C to E) were calculated for each sample shown in (B). Monocyte subsets are color coded by the corresponding pathways they demonstrated. Similar to the changes in BM monocytes after T/HS, the G pathway shows increase in MonoPC1_C2-C3 and decrease in MonoPC1_C1 compared with the M pathway. GMP: granulocyte-monocyte progenitors; MP: monocyte-committed progenitors; G-mono: G pathway-derived monocytes; MDP: monocyte-dendritic cell progenitors; cMoP: common monocyte progenitors; M-mono: M pathway-derived monocytes.

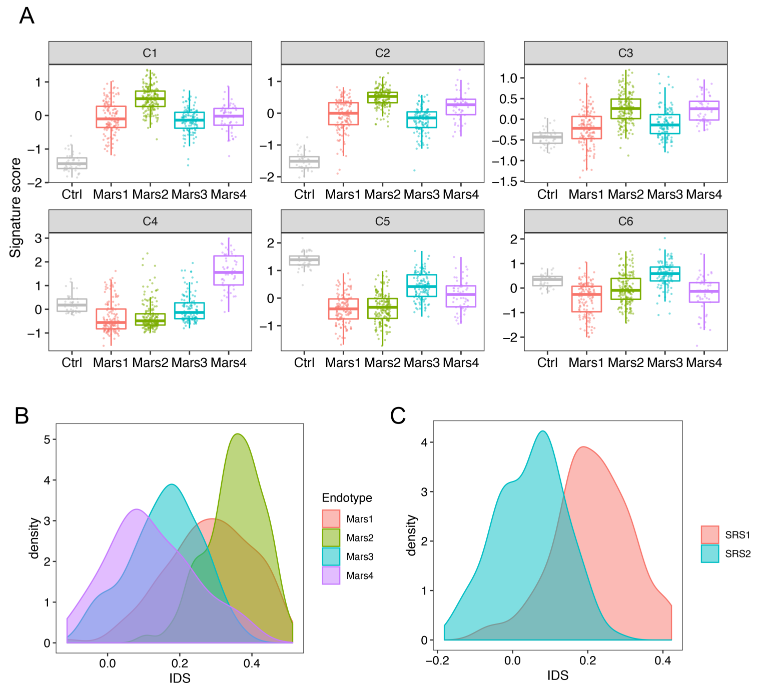

Supplemental Figure 13. Interpretation of sepsis subtypes using our findings. (**A**) Expression of the six signatures in sepsis Mars subtypes compared with healthy control. Base on Figure 13G, Mars4 displays an endotoxemia-like response. (**B**) IDS distribution among four sepsis Mars subtypes. (**C**) IDS distribution between two sepsis SRS subtypes.

Supplemental Tables:

Supplemental Table 1. Trauma Patient Clinical Characteristics

**Patient ID**	**ISS**	**Age**	**Sex**	**Injury**	**Admission chemistry**				
					SBP	HR	GCS	BD	Lactate
MM3001	22	21	Male	MVC/motorcycle	71	127	15	12	4.8
MM3005	22	35	Male	MVC/PED	60	112	3	15	7
MM3008	19	26	Male	Penetrating	70	110	15	12	6.9
MM3009	13	71	Male	Fall	88	130	15	5	5.8
MM3012	17	56	Male	MVC	125	111	14	NA	3.7
MM3015	38	21	Male	Fall	45	74	3	17	3.4
MM3016	18	32	Male	MVC	127	148	15	19	2.4
MM3020	27	78	Male	Fall	83	51	8	11	NA
MM3038	22	44	Female	MVC	112	70	3	12	5.6
MM3040	25	58	Male	Fall/TBI/SDH	138	82	3	4	22

Abbreviations:

ISS: Injury severity score; HR: Heart rate; GCS: Glasgow Coma Scale/Score; BD: Base deficit; MVC: Motor vehicle collision; PED: Pedestrian; TBI: Traumatic brain injury; SDH: Subdural hematoma; NA: Not available

Supplemental Table 2. Six signatures derived from human CD14+ monocytes

Signature Names	Genes
C1	*CLU, NKG7, S100A8, S100A9, LGALS1, S100A12, CTSD, RETN, RNASE2, PLAC8, PLBD1, FOLR3, JUN, ALOX5AP, HP, EGR1*
C2	*NAMPT, ACSL1, IL1R2, SOCS3, CD63, PIM1, CXCL8, VCAN, SLC2A3, AGFG1, CD55, SLC11A1, SAMSN1, MCEMP1, GCA, FKBP5, BCL2A1, SERPINB1, SLC25A37, CYP1B1, MCTP2, CCND3, ADM, G0S2*
C3	*FTH1, THBS1, EREG, MARCKS, SRGN, CD300E, TIMP1, CTSL, HMOX1, LITAF, CD163, GK, GLUL, HLA-DQA1, ASPH, AREG, IL1B, GK5, AQP9, PHC2, PLSCR1, GPR183, ETS2, CEBPB, CXCL2, SAP30, MAFB, FCGR1A, DDIT4, TPM4, MAP3K8, HIF1A, HLA-DRB5, ID2*
C4	*OAS2, EPSTI1, IFIT1, RNF213, PARP14, IFIT3, IFI44, XAF1, LY6E, IFI44L, MX1, ISG15, IFI6, STAT1, OAS3, IFIT2, MX2, HERC5, EIF2AK2, SAMD9L, RSAD2, APOBEC3A, OAS1, TNFSF10, GBP1, IFITM3, LAP3, TMEM123, MT2A, SP110, STAT2*
C5	*HLA-DRB1, HLA-DRA, HLA-DPA1, HLA-DPB1, FGL2, CD74, TXNIP, LGALS2, HLA-DMB, HLA-DMA, HLA-DQB1, DUSP6, CPVL, ZFP36L2, AP1S2, TGFBI*
C6	*NCF1, AHNAK, TAGLN2, CRIP1, JAML, RAB11FIP1, NUP214, LTA4H*

Supplemental Table 3. Summary of human bulk microarray datasets used in this paper

Datasets	Source	Tissue	Number of Patients	Time Points	Time-to-event data	Platform
The Inflammation and Host Response to Injury program: Trauma	GSE36809	whole blood leukocyte RNA	n = 167 severely injured patients	Within 12 h and at 1, 4, 7, 14, 21, and 28 d after the injury. The first sampled time points (8.0 ± 3.4 hrs) were used for patient classification and model training.	Time-to-recovery data were used and available from http://www.gluegrant.org/.	Affymetrix U133
The Inflammation and Host Response to Injury program: Burn	GSE37069	whole blood leukocyte RNA	n = 241	Multiple time points after burn. The first sampled time points (75.8 ± 118.0 hrs) were used for clustering and model validation.	Time-to-death data were used and available from http://www.gluegrant.org/.	Affymetrix U133
Sepsis (Mars)	GSE65682	Blood RNA	Discovery cohort (n = 263) Validation cohort (n = 216)	Only one time point: within 24h of ICU admission	Time-to-death data were used and available from GSE65682.	Affymetrix U219
Sepsis (SRS)	E-MTAB-4421	whole blood leukocyte RNA	Discovery cohort (n = 265)	First available sample taken following ICU admission	Time-to-event data were not available from public domain.	Illumina Human-HT-12 Expression BeadChips (v4)

Endotoxemia	GSE3284	whole blood leukocyte RNA	Healthy volunteers with LPS at a dose of 2 ng/kg body weight (n = 4) or 0.9% NaCl (n = 4)	Before endotoxin infusion (0 h) and 2, 4, 6, 9 and 24 hrs after infusion.	Not applicable	Affymetrix U133

References:

1. Zheng GX, Terry JM, Belgrader P, Ryvkin P, Bent ZW, Wilson R, et al. Massively parallel digital transcriptional profiling of single cells. *Nat Commun.* 2017;8:14049.
2. Amir el AD, Davis KL, Tadmor MD, Simonds EF, Levine JH, Bendall SC, et al. viSNE enables visualization of high dimensional single-cell data and reveals phenotypic heterogeneity of leukemia. *Nat Biotechnol.* 2013;31(6):545-52.
3. Singer M, Wang C, Cong L, Marjanovic ND, Kowalczyk MS, Zhang H, et al. A Distinct Gene Module for Dysfunction Uncoupled from Activation in Tumor-Infiltrating T Cells. *Cell.* 2016;166(6):1500-11 e9.
4. Gaublomme JT, Yosef N, Lee Y, Gertner RS, Yang LV, Wu C, et al. Single-Cell Genomics Unveils Critical Regulators of Th17 Cell Pathogenicity. *Cell.* 2015;163(6):1400-12.
5. La Manno G, Soldatov R, Zeisel A, Braun E, Hochgerner H, Petukhov V, et al. RNA velocity of single cells. *Nature.* 2018;560(7719):494-8.

6. Murtagh F, and Legendre PJJoC. Ward's Hierarchical Agglomerative Clustering Method: Which Algorithms Implement Ward's Criterion? 2014;31(3):274-95.

7. Mildner A, Schonheit J, Giladi A, David E, Lara-Astiaso D, Lorenzo-Vivas E, et al. Genomic Characterization of Murine Monocytes Reveals C/EBPbeta Transcription Factor Dependence of Ly6C(-) Cells. *Immunity.* 2017;46(5):849-62 e7.

8. Marigo I, Bosio E, Solito S, Mesa C, Fernandez A, Dolcetti L, et al. Tumor-induced tolerance and immune suppression depend on the C/EBPbeta transcription factor. *Immunity.* 2010;32(6):790-802.

9. Xiao W, Mindrinos MN, Seok J, Cuschieri J, Cuenca AG, Gao H, et al. A genomic storm in critically injured humans. *J Exp Med.* 2011;208(13):2581-90.

10. Butler A, Hoffman P, Smibert P, Papalexi E, and Satija R. Integrating single-cell transcriptomic data across different conditions, technologies, and species. *Nat Biotechnol.* 2018;36(5):411-20.

11. Olsson A, Venkatasubramanian M, Chaudhri VK, Aronow BJ, Salomonis N, Singh H, et al. Single-cell analysis of mixed-lineage states leading to a binary cell fate choice. *Nature.* 2016;537(7622):698-702.

12. Deneault E, Cellot S, Faubert A, Laverdure JP, Frechette M, Chagraoui J, et al. A functional screen to identify novel effectors of hematopoietic stem cell activity. *Cell.* 2009;137(2):369-79.

13. Bronte V, Brandau S, Chen SH, Colombo MP, Frey AB, Greten TF, et al. Recommendations for myeloid-derived suppressor cell nomenclature and characterization standards. *Nat Commun.* 2016;7:12150.

14. Yanez A, Coetzee SG, Olsson A, Muench DE, Berman BP, Hazelett DJ, et al. Granulocyte-Monocyte Progenitors and Monocyte-Dendritic Cell Progenitors Independently Produce Functionally Distinct Monocytes. *Immunity.* 2017;47(5):890-902 e4.
